# Surface Marker Identification to Capture Live Circulating Tumor Cells in Metastatic Triple-Negative Breast Cancer

**DOI:** 10.1158/2767-9764.CRC-25-0536

**Published:** 2026-01-15

**Authors:** Bree M. Lege, Khushali J. Patel, Brendan Panici, Ping Gong, Michael T. Lewis, Matthew J. Ellis, Chonghui Cheng

**Affiliations:** 1 Department of Molecular and Human Genetics, Baylor College of Medicine, Houston, Texas.; 2 Lester and Sue Smith Breast Center, Baylor College of Medicine, Houston, Texas.; 3 Department of Molecular and Cellular Biology, Baylor College of Medicine, Houston, Texas.; 4 Dan L Duncan Comprehensive Cancer Center, Baylor College of Medicine, Houston, Texas.; 5 Department of Radiology, Baylor College of Medicine, Houston, Texas.

## Abstract

Metastatic triple-negative breast cancer (TNBC) is highly aggressive and lacks targeted therapies. Circulating tumor cells (CTC) are invaluable for monitoring metastatic tumor progression and treatment response but are difficult to capture because of their rarity and heterogeneity. Surface-based staining for live CTCs is essential to preserve RNA quality in single cells, but current markers tend to perform poorly on more mesenchymal tumor cells such as TNBCs. To enhance live TNBC CTC detection, we developed a workflow for live CTC capture and single-cell RNA sequencing (scRNA-seq). Using a mouse model of metastatic TNBC, we identified four new CTC surface markers, AHNAK2, CAVIN1, ODR4, and TRIML2, which specifically stain tumor cells. Combining antibodies against these markers improved CTC detection rates in multiple TNBC mouse models and patient samples. Also, combining these new markers with traditional CTC surface markers enhanced detection sensitivity, achieving the highest CTC coverage. This approach identifies diverse CTC populations, while preserving RNA quality for scRNA-seq, which is essential for understanding and therapeutically targeting metastatic breast cancer. The use of these newly identified CTC markers significantly enhances both detection and live capture of CTCs, paving the way for more effective use of liquid biopsy to monitor patient prognosis and treatment response in clinical settings.

**Significance:**

CTCs are a powerful indicator of cancer metastasis; however, their scarcity makes them difficult to isolate. Current markers favor epithelial CTCs over mesenchymal populations. Our workflow for live CTC capture and sequencing enables discovery of new markers for both epithelial and mesenchymal CTCs. When combined with existing markers, we improve live CTC capture for more holistic studies of the metastatic process and offer a scalable method for discovering CTC markers.

## Introduction

Triple-negative breast cancer (TNBC) is the most aggressive breast cancer subtype, lacking targeted therapies and exhibiting the highest potential to metastasize (1, 2). Distant metastasis causes a majority of deaths in patients with breast cancer each year in the United States (2). Thus, managing metastatic TNBC progression and treatment remains a significant unmet clinical need. Circulating tumor cells (CTC) are a rare population of cancer cells that shed from primary and metastatic tumors, serving as the origin of metastatic seeding and eventually metastatic lesions (3–5). Although difficult to capture, CTCs offer a unique window of opportunity to monitor disease progression in a near “real-time” and minimally invasive manner (6, 7). Recent advancements in liquid biopsy technologies have made the enumeration of CTCs possible, with accumulating evidence indicating that CTC numbers directly correlate with disease progression and outcomes (8–10). Gene expression profiling of CTCs at the single-cell level is recognized as a promising new field for precision medicine. However, such analyses present daunting technical challenges because of the rarity of CTCs and the generally poor quality and quantity of RNA isolated from single CTCs (6). Enumeration of CTCs is typically accomplished by staining with a combination of markers which requires fixation and permeabilization, compromising RNA quality from single cells (8).

Live CTCs from a liquid biopsy can yield much higher-quality RNA sequencing (RNA-seq) data (11, 12). This requires surface-based staining for the identification and capture of live CTCs. However, many known CTC surface markers (e.g., EpCAM and HER2) are epithelial in nature and have little-to-no expression in more mesenchymal cells and tumor types, like TNBC (6, 13). The isolation of CTCs via the cell surface marker EpCAM has become common practice when performing single-cell RNA-seq (scRNA-seq) on CTCs (13). However, EpCAM alone causes a significant bias toward more epithelial CTCs as its expression is downregulated during epithelial–mesenchymal transition (EMT; refs. 14, 15). Furthermore, growing evidence suggests that a subpopulation of CTCs may lack or have lower expression of EpCAM while migrating via the bloodstream (13–15). Other studies have pointed to HER2 (or ERBB2) and EGFR (16) as additional CTC markers although HER2 is also specifically low or absent in TNBCs. EGFR may be upregulated during the transition of epithelial cells to a more mesenchymal state and this marker has shown some utility in detecting CTC populations that EpCAM enrichment alone would miss for non–small cell lung cancers (16). Moreover, the tumors from the claudin-low (CLOW) subtype of TNBC tend to show enrichment for EMT characteristics and cancer stem cell–like features (17, 18). CTCs generated by these CLOW tumors are even less likely to be recognized by CTC markers typically expressed in epithelial-type cells, leading to a high potential to be missed (18). The fundamental shortcomings of using limited CTC markers for identification are emphasized by the recent shift toward either antibody-free enrichment or combinatorial selection methods for CTCs (6, 8, 9).

CTCs are an embodiment of cancer plasticity as they repeatedly adapt to new environments over time, highlighting the need for the identification of new CTC markers that can capture more mesenchymal-like CTCs (19–22). In this study, we report the establishment of a workflow to isolate live CTCs from blood to optimize CTC capture and RNA quality. Using a metastatic TNBC mouse model, we performed single-cell RNA-seq (scRNAseq) of CTCs, generating high-quality data with detection of high numbers of genes and full coverage of the gene body. Importantly, we identified four new surface markers for live CTC detection that improve identification of TNBC CTCs. Our work provides a practical approach to studying CTCs at the single-cell level, which enables the detection of transcriptomic changes during disease progression, leading to the identification of metastasis drivers and mechanisms, and the development of prognostic tools and therapeutic targets.

## Materials and Methods

### Cell lines and transfection

All cell lines were maintained in 5% CO_2_ at 37°C and passaged every 2 to 3 days. All base medium was supplemented with penicillin and streptomycin at 1:100 (Corning, #30-002-CI). MDA-MB-231, MDA-MB231-LM2, and MCF7 cells were cultured in DMEM supplemented with 10% FBS. SKBR3 cells were cultured in McCoy 5A supplemented with 10% FBS. WHIM12 cells were cultured in DMEM/F12 (1:1) supplemented with 10% FBS, 0.1X ITS-X, 10 ng/mL EGF, 50 ng/mL 3,3′,5-triiodo-L-thyronine, and 10 ng/mL HC. 4T1 cells were cultured in RPMI-1640 with L-glutamine supplemented with 10% FBS, 10 mmol/L HEPES, 1 mmol/L sodium pyruvate, and 2.5 g/L glucose. For generating stable cell lines with fluorescent tags, lentiviral infections were performed with one of the following plasmids: EFS-GFP, pFUGW-tdtomato, and pWPT-RFP. EFS-GFP was a gift from M.T. Lewis (Addgene, plasmid #110834; ref. 23) and pFUGW-tdtomato was a gift from Connie Cepko (Addgene, plasmid #22478; ref. 24). pWPT-RFP was kindly provided by Xiang Zhang.

### *Mycoplasma* and authentication statements

All cell lines used for animal models tested negative for *Mycoplasma* before use in mice. D201-02 (Vazyme) was used according to the manufacturer’s protocol for *Mycoplasma* testing and cell lines tested negative within 1 month of use.

All cells were expanded and stored in liquid nitrogen when received. Early-passage vials within 6 months were thawed for the experiments described in this study. No further validation or authentication was performed.

### Mice

All animal procedures were performed with approval from the Institutional Animal Care and Use Committee at Baylor College of Medicine.

Spontaneous metastasis mice were generated by injecting 1 × 10^4^ LM2-GFP cells into the fourth mammary fat pad of 6- to 8-week-old NOD/SCID gamma (NSG) mice. When the primary tumors reached a volume of approximately 800 mm^3^, blood was collected via a needle puncture of the right ventricle under deep sedation, after which the animal was euthanized humanely. For CTC enrichment, the entire blood volume was passed through Parsortix (ANGLE plc) using a 4.5-μm cassette. Captured cells were stained on cassettes for 15 minutes (modified protocol with no fixation or permeabilization steps) using the negative selection marker CD45 and the nuclear marker Hoechst and subsequently harvested twice (with rinse) into RareCyte chamber slides (#24-1068-000) precoated with polyHEMA. GFP-tagged CTCs were then visualized and individually picked (40-μm needle #24-1117-000 and tube #22-1056-001) using a CyteFinder instrument (RareCyte) for scRNA-seq.

To generate tail vein mice, 1 × 10^5^ LM2-GFP cells, 1 × 10^5^ 231-TdT cells, or 5 × 10^4^ 4T1-RFP cells were injected into 6- to 9-week-old NSG mice by tail vein. Blood was collected as described above. Peripheral blood mononuclear cells (PBMC), together with CTCs, were isolated using Lymphoprep density gradient medium (STEMCELL Technologies, #7851) and ACK lysing buffer (Thermo Fisher Scientific, #A1049201). Cell pellets were stored in 10% DMSO in FBS at −80°C until used for immunofluorescent (IF) staining or flow cytometry.

For patient-derived xenograft (PDX) mouse samples, blood for PDX models was collected when tumors were approximately 0.5 g in weight. Blood was collected either via a needle puncture of the right ventricle under deep sedation or from the inferior vena cava, after which the animal was euthanized humanely. PBMCs were isolated and stored as described for tail vein mice.

For control PBMCs (mouse), blood was collected from normal female mice and processed as described above for tail vein mice. Isolated PBMC pellets were either used immediately or stored in 10% DMSO in FBS at −80°C until used for IF staining or flow cytometry.

### Human samples

Human studies were conducted in accordance with and approved by the institutional review board (IRB) and in accordance with the ethical guidelines as laid out in U.S. Common Rule.

For control PBMCs (human), buffy coats from healthy human donors were obtained from Gulf Coast Regional Blood Center. PBMCs were isolated and stored at −80°C as described above until use.

For CTC analysis, blood was obtained from de-identified patients with metastatic TNBCs after receiving their written informed consent as per IRB protocol. In EDTA blood draw tubes, 6 to 8 mL of peripheral blood was collected and processed by lymphoprep as described above. Isolated PBMC fractions were spread onto slides and stored at −80°C until staining.

### scRNA-seq

Single cells were visualized in chamber slides coated with polyHEMA for isolation and deposited in tubes via a RareCyte aspirating needle in 400 to 600 nL PBS. Individual tubes containing deposited cells were processed using SMARTseq2 protocol as previously described (25) with several modifications. Shortly, 2 μL of lysis buffer was added to each tube, consisting of Triton, RNase inhibitor, dNTPs, and Oligo dT, and incubated first at room temperature for 5 minutes and then at 72°C for 5 minutes. Reverse transcription mix was added (3 μL), containing 5x first-strand buffer, RNase inhibitor, dithiothreitol, MgCl_2_, betaine, template switching oligo, and SMARTScribe reagent. Samples were incubated at 42°C for 90 minutes and then at 70°C for 5 minutes. The amplification cycle number was determined for each set of cells using iTaq SYBR green reagent (Bio-Rad, #1725121) and run for 40 cycles. Amplification mix, containing IS PCR primer, KAPA HiFi hotstart, and Lambda exonuclease, was added (7.5 μL) and amplification was run at ∼28 to 35 cycles depending on the single cell. A detailed list of reagents is provided in Supplementary Table S1.

### Library prep/sequencing

Libraries were prepared for sequencing from amplified cDNA using tagmentation via the Nextera XT Library Prep Kit (library kit FC-131-1096 and index kit FC-131-1001) and standard kit protocol. Libraries were pooled and sequenced on a NextSeq 550 platform. Data were demultiplexed using Basespace BCL Convert v1.2.1.

### Data analysis

RNA-seq reads were aligned to the human genome (GRCh38, primary assembly) and transcriptome (GENCODE version 38, primary assembly) using STAR version 2.7.10a with the following parameters: -outFilterMultimapNmax 1 -outSAMstrandField intronMotif -outFilterType BySJout -alignSJoverhangMin 8 -alignSJDBoverhangMin 2 -alignEndsType Local -limitOutSJcollapsed 8000000 -quantMode GeneCounts -soloType SmartSeq -soloUMIdedup Exact -soloStrand Unstranded -outFilterScoreMinOverLread 0.3 -outFilterMatchNminOverLread 0.3 -outReadsUnmapped Fastx. Preliminary filtering based on STAR alignment log files eliminated three CTCs which had less than 50% uniquely mapped reads. Additionally, one CTC which had more than 50% of reads from mitochondrial genes was also filtered out. Mitochondrial percent was assessed by using Seurat PercentageFeatureSet (). The 37 remaining CTCs were used for all downstream analysis. Six cell culture samples, which passed both above filters, were used as baseline controls. All plots throughout were generated with ggplot2 unless otherwise specified, all heatmaps were generated with the R package Pheatmap, and the majority of the analyses were performed in R unless otherwise specified.

#### Figure 1 quality check analysis

(B and C) STAR log files provided uniquely mapped read percent information for each cell. STARsolo output was read into R using the Read10X() function, and a Seurat object was created using min.cell = 5 and min.features = 1,000 which did not filter out any cells but filtered out genes which were expressed by less than five of the cells. Feature count obtained from Seurat metadata and PercentageFeatureSet() was used on the Seurat object to determine the percentage of reads attributed to mitochondrial genes, and dot plots were generated using these features. (D) Count data for CTCs and LM2 cell culture, normalized using DESeq2, a sample-wise spearman correlation matrix [cor (x, method = “spearman”)] were obtained by using the normalized gene expression for all samples and this was used to generate a correlation heatmap. The dendrogram is shown in Supplementary Fig. S1B. (E) Data for GSE112845 and GSE94820 were obtained from the Gene Expression Omnibus (GEO) Dataset database. Data for GSE164898 were obtained from the 10X genomics dataset portal. Gene numbers were obtained from Seurat for each dataset and CTC data, and ggplot2 was used to generate comparison plot. (F) Cancer Cell Line Encyclopedia (CCLE) cell line data (select breast cancer cell lines from BioProject PRJNA523380) were obtained using SRA tools prefetch and fasterq-dump. STAR v2.7.10a, GRCh38 primary assembly, and gencode v38 primary assembly were used to align RNA reads for GSE164898 and CCLE data. geneBody_coverage.py from RSeQC was run for CTC, LM2, GSE164898, and CCLE data to generate gene body coverage plots, and means were taken for each dataset to generate the final plot. (G) BAM files generated for previous plots were used to visualize coverage for individual genes in Integrative Genomics Viewer.

#### Figure 2 known marker analysis

(A) Data from CTCs normalized by size factor with DESeq2 (test = “LRT”, reduced = ∼1, useT = TRUE, minmu = 1e−6, and minReplicatesForReplace = Inf as recommended for single-cell data) and log_2_ transformed were used with ggplot2 to produce a boxplot of gene expression for the three previously published CTC markers. (B) CCLE expression and proteomics data were obtained from the DepMap portal. Subtypes were distinguished {claudin low as defined in Heiser and colleagues (CCLE; ref. 26) and Fougner and colleagues [The Cancer Genome atlas (TCGA); ref. 27]} and split, and ggplot2 was used to produce boxplot representation of protein expression. (C) TCGA breast cancer data were obtained using GDCquery() and GDCprepare() in R. Subtypes were distinguished and split to produce boxplot representation of RNA expression [transcripts per million (TPM)]. CPTAC expression and proteomics data were obtained from cBioPortal and used to produce boxplot representation of protein expression for potential new markers.

#### Figure 3 new marker analysis

Count data for CTCs (24,803 genes after eliminating all rows with rowMeans = 0), normalized by size factor with DESeq2, were used for comparison with Human Protein Atlas (HPA) single-cell data for PBMCs downloaded from HPA v20.1, Ensembl v92.38. All PBMC-expressed genes (count >0) were subtracted, leaving 12,483 genes. Additionally, GTEx whole-blood expression data were obtained from the GTEx portal and genes with GTEx expression >15 were filtered out, leaving 12,271 genes. Next, genes with low expression (normalized average expression <8) and genes expressed by less than 30% of the CTCs were filtered out, leaving 569 genes and 505 genes, respectively. Using genebiotype = “protein_coding” from the BioMart database eliminated genes that were not protein-coding genes, leaving 304 genes. Genes predicted to be on the cell surface using HPA gene location designation for plasma membrane were selected, leaving 35 genes. Lastly, published IF images for these 35 proteins were manually assessed and genes with little to no surface staining or having no antibody available at the time of assessment were eliminated, resulting in 14 new candidate genes plus *EGFR*. (B) Count data from CTCs, normalized with DESeq2 and log transformed, were used with ggplot2 to produce boxplots of gene expression for candidate genes. (C) Data for GSE180097 were obtained from the GEO dataset database, and ggplot2 was used to produce boxplot for expression of potential new markers. (D) CCLE expression and proteomics data were used to produce boxplot representation of protein expression for potential new markers. (E) CPTAC expression and proteomics data were used to produce boxplot representation of protein expression for potential new markers.

### Immunofluorescence

For all adherent cell lines, cells were detached using 0.05% trypsin, rinsed with PBS, and settled on poly-L-lysine–coated slides for at least 45 minutes at 37°C before fixation. Fixation was performed for 10 minutes at room temperature in PBS 1X containing 4% paraformaldehyde. Cells were blocked for at least 1 hour in PBS/2% BSA and incubated in primary antibody overnight at 4°C. Following three washes in PBS, samples were incubated with the respective Alexa Fluor (AF)–coupled secondary antibodies for 1 hour at room temperature. For staining with any primary AF-conjugated antibodies plus DAPI, samples were incubated at room temperature for 2 hours before rinsing and mounting. For cytokeratin staining, samples were permeabilized at room temperature for 10 minutes in PBS/0.1% Triton before blocking and staining overnight with cytokeratin, followed by secondary antibody staining at room temperature for 1 hour. Permeabilization was only performed after all surface staining was finished. After three additional washes in PBS, samples were mounted with ProLong Diamond Antifade Mountant.

For PBMC samples (including control human/mouse and mouse tail vein), pellets were used fresh after Lymphoprep processing or thawed from −80°C storage and rinsed with PBS. Cells were settled on poly-L-lysine–coated slides for at least 45 minutes at 37°C before fixation. For all staining involving PBMCs, fixation was performed for 20 minutes at room temperature in 10% neutral buffered formalin. Fixed slides were placed in the TiYO Autofluorescence quenching system with PBS 1X covering surface for 30 minutes to reduce autofluorescence during imaging. Spike-in samples were prepared from control PBMC samples mixed with cells from cell line preparations and slides were prepared as above. Cells were blocked for at least 3 hours in 5% goat serum (GS) and incubated in primary antibody overnight at 4°C. Following three washes in PBS, samples were incubated in PBS/2% GS containing the respective AF-coupled secondary antibodies for 1 hour at room temperature. For staining with any primary AF-conjugated antibodies plus DAPI, samples were incubated at room temperature for 2 hours before rinsing and mounting in media. For samples stained with cytokeratin, samples were permeabilized at room temperature for 10 minutes in PBS/0.1% Triton before staining overnight with cytokeratin, followed by secondary staining at room temperature for 1 hour. Permeabilization was only performed after all surface staining was finished. After three additional washes in PBS, samples were mounted with ProLong Diamond Antifade Mountant.

For all PDX samples, pellets were frozen after Lymphoprep processing, thawed from −80°C storage, and rinsed with PBS. Pellets were resuspended in Transfer Fluid (RareCyte) for 5 to 10 minutes at room temperature, spread on slides, and then allowed to dry for 30 minutes. Fixation and staining were performed as described above for PBMC samples, except DAPI was not included. As an alternate nuclear marker, hnRNPM antibody was tagged with Qdot625 (Thermo Fisher Scientific, #S10452) and added as the last staining step for 2 hours at room temperature.

For all patient samples, pellets were used fresh after Lymphoprep processing. Pellets were resuspended in Transfer Fluid for 5 to 10 minutes at room temperature and spread on slides. Slides were allowed to dry for 30 minutes and then stored at −80°C until staining. Fixation and staining were performed as described for PBMC samples. A full list of antibodies is provided in Supplementary Table S2.

All images were taken at 40X using RareCyte imager software and CytePicker I platform.

### Image analysis

ImageJ [ImageJ 1.52q; Java 1.8.0_172 (64-bit)] macros were run on pictures of individual color channels taken at the same location for each location analyzed. The generated readings for all channels, in each location, were assembled as a matrix for filtering and counting positive cells in each population assessed (i.e., cell line or PBMCs). CTCs from tail vein blood samples were analyzed manually.

#### Figure 4B, Supplementary Fig. S4B and S4C quantifying staining of individual markers or cocktails on human PBMCs

DAPI signal was used create a mask to define cell locations, and CD45 and GFP signals were used to define cells positive for CD45 and negative for GFP as unambiguously immune cells and then signal in the marker channel was assessed as positive or negative to determine percent positive staining on PBMCs. Mean gray value was used and cutoffs were uniformly applied across all pictures after visual confirmation of detectable signal. Pictures assessed *n* = 4 to 5 for each marker.

#### Figure 4D and F quantifying staining of individual markers or cocktails on cell lines

DAPI signal was used create a mask to define cell locations, and then signal in the marker channel was assessed as positive or negative to determine percent positive staining on cells. Mean gray value was used and cutoffs were uniformly applied across all pictures after visual confirmation of detectable signal. Pictures assessed for individual markers: *n* = 4 for MCF7, SKBR3, and LM2 and *n* = 5 to 15 for WHIM12, with minimum of 79 cells counted for each cell line. Pictures assessed for cocktails: *n* = 4 to 6, with minimum of 90 cells counted for each cell line.

#### Figure 5B and E quantifying staining on CTCs in blood from tail vein mice

DAPI signal was used to define cell locations. CD45 and TdTomato/RFP signals were used to define cells positive for TdTomato/RFP and negative for CD45 as CTCs, and then signal in the marker channel was assessed as positive or negative to determine percent positive staining on CTCs. Quantification was done manually. Pictures assessed for 231 blood samples for each cocktail were *n* = 5 to 9, with minimum of 160 CTCs counted. Pictures assessed for 4T1 blood samples for each cocktail were *n* = 10, with minimum of 136 CTCs counted. Significance was determined by one-way ANOVA.

### Flow cytometry

For all adherent cell lines, cells were detached using 0.05% trypsin and rinsed with PBS 1X with 2% FBS. For all PBMC samples, including control human/mouse and mouse tail vein samples, pellets were used fresh after Lymphoprep processing or thawed from −80°C storage and rinsed with PBS/FBS. For all staining, cells were suspended in PBS/FBS. Cells were stained and run same day, so no fixation was used. Unconjugated antibodies for AHNAK2, CAVIN1, ODR4, and TRIML2 were tagged with the Lightning-Link Alexa Fluor 488 Fast Conjugation kit (Abcam, ab236553). Samples were incubated with primary-conjugated antibodies (EpCAM, HER2, and EGFR in both AF488 and AF647 and CD45 in PE) at 4°C for 30 to 45 minutes and rinsed with PBS/FBS twice. UltraComp eBeads (Invitrogen, #01-2222-42) and/or unstained controls were used to set baseline voltage. When possible, corresponding untagged cell lines were used for unstained controls of GFP-, TdTomato-, or RFP-tagged cells. Single-color controls (cells and beads) were used for each channel to finalize voltage settings and for compensation of color overlap. Cell line samples were run on Beckman Coulter CytoFLEX. Tail vein PBMC samples were run on BD LSRFortessa. FlowJo software v10 was used for analysis. Significance was determined by a paired Student *t* test (two-tailed). A full list of antibodies is provided in Supplementary Table S2. RRIDs for cell lines, plasmids, and software are provided in Supplementary Table S3.

## Results

### Development of a workflow for scRNA-seq of CTCs from a TNBC mouse model

**Figure 1.**
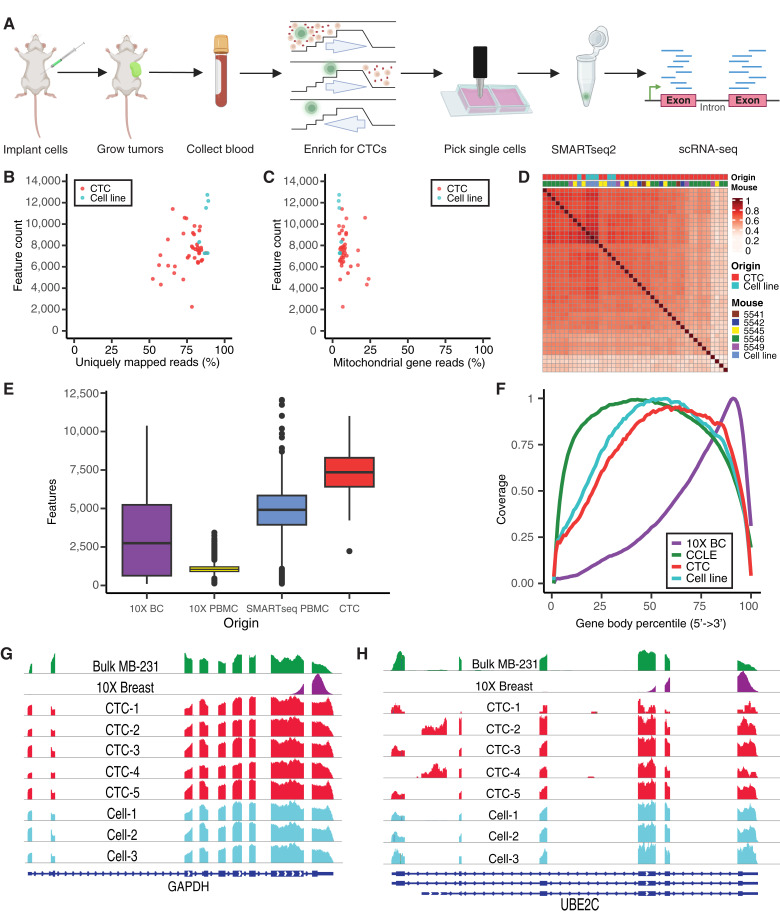
CTC isolation and processing workflow for high-quality scRNA-seq. **A,** CTC isolation workflow consisting of tumor implantation, collection of terminal blood sample after 8 to 9 weeks, Parsortix PR-1 processing, CTC visualization/picking using GFP signal, and scRNA-seq via SMARTseq2. CTCs were generated using the TNBC model LM2. **B** and **C,** Scatter plot of gene count vs. percent of uniquely mapped read (ρ = 0.3398; **B**) or percent of mitochondrial read (ρ = −0.2776; **C**) for LM2 CTCs (red; *n* = 37) and cell line (cyan; *n* = 6). ρ represents the value of the Spearman correlation coefficient. **D,** Heatmap displaying Spearman correlation using all genes with hierarchical clustering for LM2 CTCs (red) and cell line (cyan). **E,** Boxplots showing gene counts for four single-cell datasets: Breast Cancer 10X (purple), PBMC 10X (yellow), PBMC SMARTseq (blue), and LM2 CTCs SMARTseq (red). Boxes indicate median and IQR; whiskers show minima and maxima, and dots indicate outliers. **F,** Meta-coverage plot showing all reads across gene body coverage for all features for four datasets: Breast Cancer 10X single-cell data (purple), 14 CCLE breast cancer lines (green), LM2 CTCs (red), and LM2 cell line (cyan). See Supplementary Fig. S1B–S1D for plots of individual samples within each dataset. **G** and **H,** Coverage plots showing gene structure and coverage for GAPDH (**G**) and UBE2C (**H**) in bulk MDA-MB-231 (green), combined single-cell data for Breast Cancer 10X (purple), five individual LM2 CTCs (red), and three individual LM2 cell culture cells (cyan). Blue tracks at the bottom indicate RefSeq annotations. BC, breast cancer. [**A,** Created in BioRender. Bobkov, G. (2026) https://BioRender.com/k55lcd1].

To identify cell surface markers presented in TNBC CTCs, we used an experimental TNBC metastasis mouse model and formulated a workflow that allows collection and scRNA-seq of live CTCs (Fig. 1A). We used the well-established metastatic human TNBC model MDA-MB231-LM2 (LM2) labeled with GFP. We injected the LM2 tumor cells in the mammary fat pad, allowing for primary tumor growth and spontaneous metastasis. Once the tumors reached terminal volume (800–1,500 cm^3^), whole blood was collected and processed using microfluidic size-based enrichment of CTCs, which were output directly into chamber slides in PBS 1X for visualization. Next, fluorescently tagged CTCs were individually picked from chamber slides coated with polyHEMA and processed via a modified SMARTseq2 protocol (25) for RNA-seq and analysis. For sequencing, 41 CTCs were isolated from five LM2 tumor-bearing mice. As a positive control for scRNA-seq quality, six LM2 cells grown in tissue culture (denoted as LM2 cell line) were processed in conjunction with CTCs. After filtering out cells with less than 50% unique mapping rate and greater than 50% mitochondrial reads, the remaining 37 CTCs and six cultured LM2 cells were used for further analysis. Each cell had a uniquely mapped read depth ranging from 0.6 to 2.9 million with an average of approximately 1.8 million.

The median detection was 7,353 genes (range, 2,227–11,010 genes) per CTC, and the CTCs exhibited a similar mapping rate and gene detection as the LM2 cell line cells, indicating that transcriptome coverage remains high across the CTC dataset (Fig. 1B; Supplementary Fig. S1A). The mitochondrial gene reads contributed less than 30% and did not correlate with the number of detected genes (Fig. 1C), indicating that the reads contributed by mitochondrial genes in several of the CTCs do not limit the number of genes detected, and may reflect the biology of the CTCs. This is supported by previous findings that subpopulations of cells expressing higher mitochondrial read counts are part of the biological function of those cells (28–32).

Assessing the global gene expression of CTCs indicates that CTCs did not cluster based on donor animal (Fig. 1D; Supplementary Fig. S1B) and most of the CTCs displayed strong correlations to each other and the cells grown in tissue culture (Fig. 1D). Some CTCs had low correlation with the other cells despite passing quality metrics, suggesting that these CTCs have unique transcriptional profiles. These results show that CTCs are heterogeneous in nature, yet share similarities across different mice, supporting an underlying biological consistency in the metastatic process among the CTCs from different mice.

The selection of a sequencing technology that captures the necessary depth and detail to study a rare cell population at scale is also critical. However, inherent limitations in sequencing technologies lead to diminishing returns when sequencing beyond a certain depth. Most scRNA-seq datasets are generated using 10X chemistry because of its high-throughput capacity. SMARTseq2 chemistry, on the other hand, enables higher gene detection than 10X single-cell chemistry (33), which is crucial for in-depth molecular characterization. As an example, existing 10X scRNA-seq datasets generated from breast cancer samples (GEO164898) and from PBMCs (GEO112845) showed an average gene count per cell of just more than 2,500 and 1,000, respectively (Fig. 1E). However, a SMARTseq dataset generated from PBMCs (GEO94820) showed an average of close to 5,000 genes per cell, almost five times greater than the 10X data generated from a similar source material. Our CTC data have an average above 7,300 genes per cell. The increased average gene detection using SMARTseq2 chemistry improves our ability to more comprehensively characterize gene expression at the single-cell level. Another advantage of SMARTseq2 chemistry is the coverage across the length of the gene body (25, 34), which allows for more accurate gene detection during alignment and transcript-based analysis. Gene body or meta-coverage plots are intended to check whether read coverage is uniform and whether there is any 5′ or 3′ bias in the data. Although the 10X scRNA-seq datasets show a 3′ bias, the gene body coverage for our SMARTseq2-based CTC data showed coverage from 5′ to 3′ similar to that from bulk RNA-seq, without any 5′ or 3′ bias (Fig. 1F and G; Supplementary Fig. S1C–S1E).

In addition to profiling gene expression, our SMARTseq2 CTC dataset also enabled the detection of RNA isoforms. For instance, we detected a differential usage of an alternative transcription start site of the ubiquitin-conjugating enzyme E2 C (UBE2C) in some CTCs as compared with the LM2 cell line (Fig. 1H). UBE2C transcripts with the alternative transcription start site generate a shorter isoform, UBE2C-s, which eliminates the first 39 amino acids encoded by an intrinsically disordered region, previously proposed to mediate protein–protein interaction. The existence of UBE2C-s only detected in CTCs suggests a possibility of UBE2C-s isoform involved in CTC properties, which warrants future investigations. The detail of the SMARTseq2 single-cell sequencing displays differences, which would not be apparent in either bulk sequencing or 10X single-cell data. Together, our results show that our workflow is optimized for isolation of live CTCs, enabling detailed analysis of CTC scRNA-seq.

### Current CTC surface markers are biased toward epithelial cells

**Figure 2.**
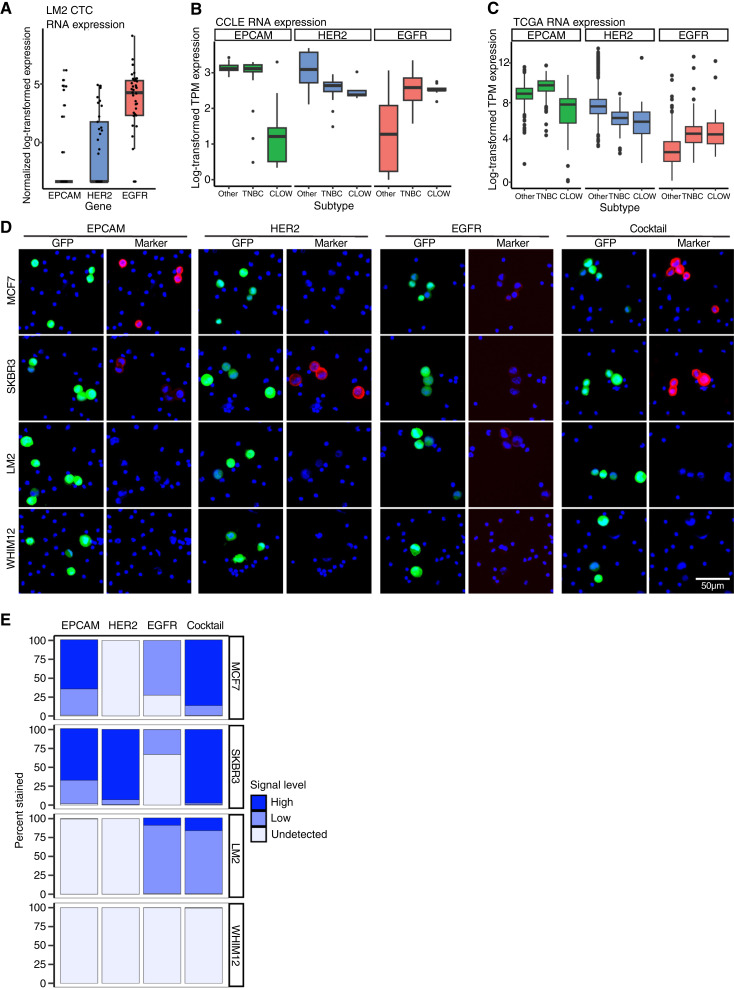
Current surface markers bias CTC identification toward epithelial tumor cells. **A,** Normalized and log_2_-transformed RNA expression for *EPCAM*, *HER2*, and *EGFR* from scRNA-seq of LM2 CTCs (*n* = 37). **B** and **C,** Log_2_-transformed TPM expression for *EPCAM*, *HER2*, and *EGFR* in CCLE Breast Cancer cell lines (**B**) and tumors from patients with breast cancer from TCGA (**C**). Samples were divided into claudin-low (CLOW), TNBC minus CLOW, and all other subtypes. Boxes indicate median and IQR; whiskers show minima and maxima and dots indicate outliers. **D,** IF staining of EpCAM, HER2, and EGFR individually and together in a cocktail (red; right column for each marker) on GFP-labeled breast cancer cell lines spiked into human control PBMCs. DAPI (blue) as a nuclear counterstain. **E,** Flow cytometry quantification of staining for EpCAM, HER2, and EGFR individually and together (Cocktail) on MCF7, SKBR3, LM2, and WHIM12 cell lines. See Supplementary Fig. S2D and S2E for histogram gates.

EpCAM, HER2, and EGFR are the most commonly used surface markers for breast cancer CTCs and form a good foundation for the detection of CTCs (12). However, they may not be sufficient to capture the entirety of the highly diverse CTC population. Assessing their expression in our LM2 CTC scRNA-seq dataset revealed that none of the three markers are expressed in every CTC (Fig. 2A). *EPCAM* is detected in eight of 37, *HER2* is in 17 of 37, and *EGFR* is by far the most detected at 33 of 37 CTCs. In fact, three of 37 CTCs do not express any of these three markers (Supplementary Fig. S2A) and thus would fail to be detected by even a cocktail of these markers.

To determine whether the known markers have variable coverage across different breast cancer subtypes, we analyzed their expression in the CCLE (35) breast cancer dataset. We divided the CCLE cell lines to non-TNBC (denoted as other), TNBC (excluding CLOW), and the highly mesenchymal CLOW subtype (Fig. 2B; Supplementary Fig. S2B). Although TNBC cell lines expressed *EPCAM*, CLOW cell lines displayed drastically reduced *EPCAM* expression at both the RNA and protein levels. As expected, both TNBC and CLOW cell lines have lower *HER2* expression than the other subtypes. *EGFR* expression is higher in TNBC than other subtypes, but the CLOW cell lines show lower expression as compared with TNBC. Analysis of primary tumors from patients with breast cancer in the TCGA and CPTAC (36) datasets displayed similar trends for these markers (Fig. 2C; Supplementary Fig. S2C and S2D), indicating the variable abilities of existing markers to capture CTCs based on breast cancer subtypes.

Interestingly, *HER2* is detected at the RNA and protein levels in the CCLE, TCGA, and CPTAC TNBC samples (Fig. 2B and C; Supplementary Fig. S2B–S2D), as well as in our LM2 CTC scRNA-seq dataset (Fig. 2A). As TNBC is defined as HER2 negative, we speculated that its expression may not be visible via IF staining of CTCs. Thus, we tested the surface expression levels of HER2, as well as EpCAM and EGFR, by staining four breast cancer cell lines with these markers individually, as well as in a cocktail. To mimic CTC samples, these cells were GFP tagged and mixed with control human PBMCs prior to staining. EpCAM showed strong staining in the luminal MCF7 cells and dim staining in the HER2^+^ SKBR3 cells but was not visually detectable in the TNBC LM2 and WHIM12 cells (Fig. 2D). HER2 was only detected in the SKBR3 cells, whereas EGFR had low/dim signal across three of four cell lines, with WHIM12 again showing no signal (Fig. 2D). When these three markers were combined into a cocktail, they showed strong signal and near 100% coverage for MCF7 and SKBR3, reasonable coverage but almost invisible signal for LM2, and no coverage for WHIM12 cells (Fig. 2D). Flow cytometry staining of these cell lines showed similar results (Fig. 2E; Supplementary Fig. S2E and S2F). EpCAM contributes the majority of signal for MCF7, HER2 contributes the majority signal for SKBR3, and although very low, EGFR contributes the majority signal for LM2. However, WHIM12 remains undetected using these markers. These findings show the inherent limitations of the existing CTC surface markers in detecting TNBC cells and underscore the need to expand markers to capture the full heterogeneity of CTCs.

### Identification of new surface proteins for CTC detection

**Figure 3.**
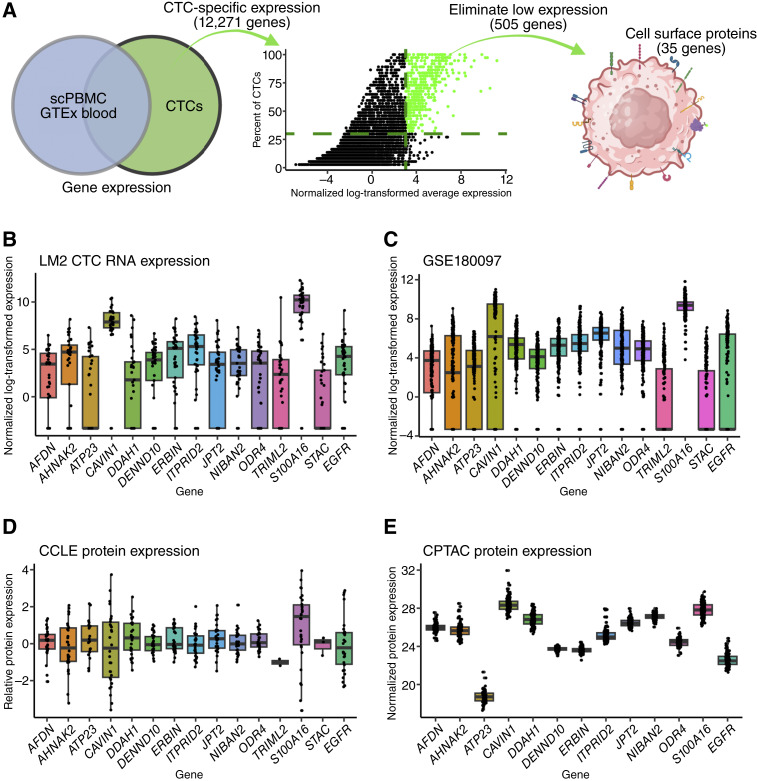
scRNA-seq analysis identifies potential CTC surface markers. **A,** CTC surface marker analysis workflow, including steps to subtract genes found in RNA-seq datasets for scPBMC and GTEx blood, filter out low CTC expression genes, and select for predicted cell surface protein-coding genes. **B** and **C,** Normalized log_2_-transformed expression of 14 potential CTC surface markers and known marker *EGFR* in LM2 CTCs (*n* = 37; **B**) and mouse and human breast cancer CTCs from GSE109761 (*n* = 241; **C**). **D** and **E,** Relative protein expression of 14 potential CTC surface markers and known marker *EGFR* in CCLE Breast Cancer cell lines (**D**) and normalized protein expression in tumors from patients with breast cancer from CPTAC (**E**). Boxes indicate median and IQR; whiskers show minima and maxima, and dots indicate outliers. BC, breast cancer. [**A,** Created in BioRender. Bobkov, G. (2026) https://BioRender.com/k55lcd1].

To identify new markers for mesenchymal-like TNBC CTCs, we used our scRNA-seq data of CTCs generated from LM2 spontaneous metastasis (Fig. 3A). First, we removed genes detected in CTCs that are also expressed in immune cells by utilizing publicly available datasets for scRNA-seq on PBMCs (37) and GTEx (RRID: SCR_001618) whole-blood sequencing. Second, the remaining CTC-specific genes (12,271 genes) were further filtered based on their expression level and coverage in CTC samples. We selected genes in the top 5% of averaged expression across CTCs and eliminated genes expressed by less than 30% of the CTCs. This resulted in 505 gene candidates. Third, protein-coding genes that were previously reported to be localized to the cell surface (38) were selected for further assessment. The 35 cell surface proteins were further screened for reported evidence of cell surface localization and antibody availabilities. This selection process resulted in a final list of 15 surface marker candidates, which included the known CTC marker EGFR, supporting the promise of this approach.

Overall, the RNA expression of these potential markers was variable in our LM2 CTC dataset, with *CAVIN1* and *S100A16* having the highest average expression (Fig. 3B). Unsurprisingly, none of the markers, including *EGFR*, were expressed by every single CTC, but each was expressed by at least 45% of the CTCs. To assess whether these markers were broadly applicable to breast cancer CTCs, we analyzed their expression in a publicly available scRNA-seq dataset (12) consisting of CTCs from two mouse models (LM2 and BR16), as well as patient samples (Fig. 3C; Supplementary Fig. S3A–S3C). Most of the markers showed good coverage and expression when CTCs from all three sources were pooled (Fig. 3C). Separating these samples by the source of CTCs demonstrates the variation of expression based on the CTC source and detection method (Supplementary Fig. S3A–S3C). CTCs from the previously published LM2 model, also isolated by a fluorescent tag, express these markers in a very similar pattern to our LM2 CTCs (Fig. 3B; Supplementary Fig. S3A). In contrast, CTCs from the BR16 model, which was derived from CTCs from a patient with hormone receptor–positive breast cancer, showed greater differences in expression (Supplementary Fig. S3B). For example, BR16-derived CTCs were almost completely negative for *EGFR*, which is consistent with our data showing that non-TNBC cells have much lower *EGFR* expression (Fig. 2B–E). The patient CTCs originated from 30 patients with mixed subtypes, with only four of 30 being TNBC, which were identified via an EpCAM/HER2/EGFR cocktail, also showed lower average *EGFR* expression (Supplementary Fig. S3C). The expression of these candidate CTC markers was further analyzed in the CCLE, TCGA, and CPTAC datasets at the RNA and protein levels (Fig. 3D and E; Supplementary Fig. S3D–S3F). The expression variability among these markers again emphasizes the need for combinatorial approaches. Together, the surface markers nominated from our CTC scRNA-seq are expressed across breast cancer models and subtypes.

### New surface markers expand detection of mesenchymal tumor cells

**Figure 4.**
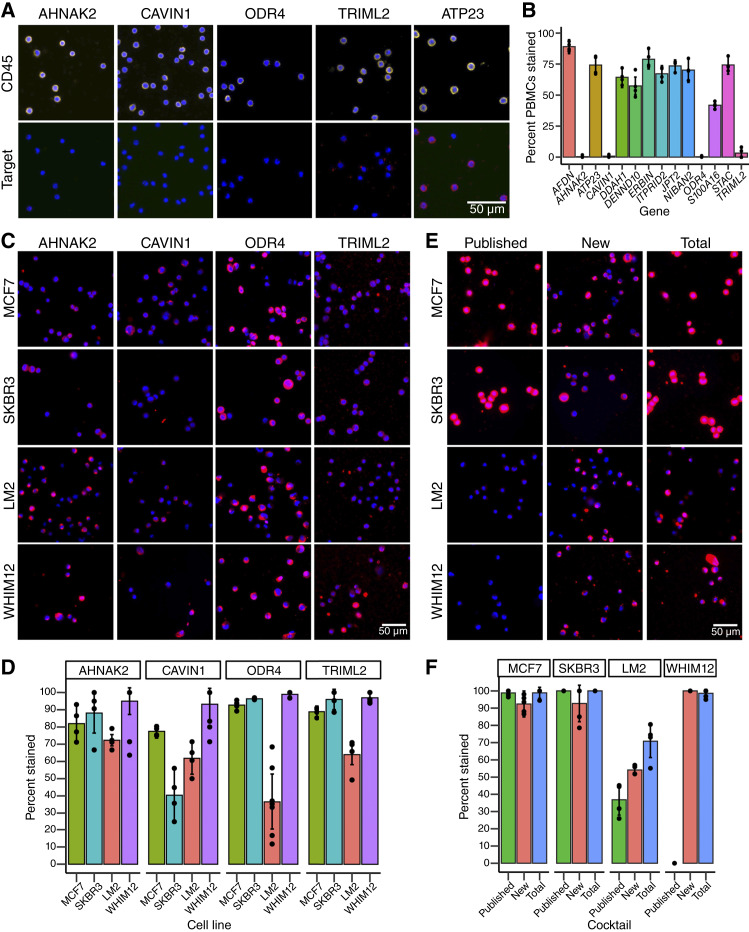
New surface markers improve mesenchymal cell detection. **A,** Representative images of human PBMCs stained with CD45 (top; yellow) and CTC surface marker candidates (bottom; red). Four markers with little or no PBMC staining and one marker with strong PBMC staining (ATP23) are shown. **B,** Quantification of percent positive staining for 14 individual markers on human PBMCs. See Supplementary Fig. S4A for representative images. **C** and **D,** IF staining (**C**) and quantification of percent positive staining on cell lines (**D**) for four selected surface markers (red). **E** and **F,** Representative images (**E**) and quantification of percent positive staining on cell lines (**F**) for three surface marker cocktails (red): Published (EpCAM, HER2, and EGFR), new (AHNAK2, CAVIN1, ODR4, and TRIML2), and total (EpCAM, HER2, EGFR, AHNAK2, CAVIN1, ODR4, and TRIML2). DAPI (blue) served as a nuclear counterstain, and individual markers or cocktails in red. For quantification in **B**, **D**, and **F**, data are represented as mean ± SD, with *n* > 4 images analyzed per condition.

Two key considerations in selecting new CTC markers are minimizing off-target staining of the surrounding PBMCs and maximizing the capture of diverse CTC populations. To eliminate any markers with high off-target staining, human PBMCs from healthy individuals were stained with the 14 candidate markers, along with the immune cell marker CD45 as a positive control (Fig. 4A and B; Supplementary Fig. S4A). Ten of these 14 new markers showed positive staining on PBMCs and were eliminated from further consideration (Supplementary Fig. S4B). The remaining four markers, AHNAK2, CAVIN1, ODR4, and TRIML2, showed minimal positive staining on PBMCs (Fig. 4B) at levels comparable with those observed with the three previously published CTC markers (Supplementary Fig. S4B). These four markers were further analyzed for their expression in different breast cancer subtypes and PBMC data (Supplementary Fig. S4E).

We performed IF staining of these four candidates on the following breast cancer cell lines: MCF7 (estrogen receptor positive), SKBR3 (HER2+), LM2 (TNBC), and WHIM12 (TNBC, Fig. 4C). The candidate surface markers ranged in coverage from approximately 36% to 99%, with at least one marker having greater than 70% coverage in all cell lines (Fig. 4D). Importantly, they were not biased toward epithelial or mesenchymal subtypes, unlike the epithelial bias of the currently used surface markers (Fig. 2). AHNAK2 and TRIML2 have a consistently high rate of coverage across cell lines, ODR4 has a consistently high signal but greater variation in coverage, and CAVIN1 has both variable coverage and signal intensity (Fig. 4C and D). The most striking results are from WHIM12 cells, which were completely undetected by the published markers (Fig. 2D and E) but had near 100% coverage with each of the new markers (Fig. 4D). This broad coverage suggests that the four new markers have a high potential to expand the detection of CTCs to a wider variety of subpopulations.

To enhance the coverage of heterogeneous cell populations beyond any individual marker, the four new markers, AHNAK2, CAVIN1, ODR4, and TRIML2, were combined into a cocktail termed “New” cocktail. As a comparison, the known markers EpCAM, HER2, and EGFR were combined into a “Published” cocktail. Finally, we combined the four new and three published markers into a “Total” cocktail to maximize staining intensity and coverage. Of note, combining these markers into cocktails did not introduce false-positive staining on PBMCs (Supplementary Fig. S4C). Next, the four breast cancer cell lines used above were stained via IF with the three cocktails to assess differences in positive staining (Fig. 4E and F). Quantification of the staining results revealed that the coverage for MCF7 and SKBR3 cells was between 90% and 100% with minimal differences between all three cocktails (Fig. 4F). In contrast, the published cocktail stained less than 40% of LM2 cells, and the signal was very weak (Fig. 4E and F; Supplementary Fig. S4D). Signals of the positive staining were often not visually apparent without using settings that enhance signal saturation well above the level used for MCF7 and SKBR3 cells (Supplementary Fig. S4D). The new cocktail enhanced detection of LM2 cells to 60% and the total cocktail further enhanced signal intensity and expanded coverage to more than 70% for LM2 cells (Fig. 4E and F). Similarly, the published cocktail did not stain WHIM12 cells, but the new and total cocktails had almost complete coverage (Fig. 4E and F; Supplementary Fig. S4D). Collectively, these results indicate that the four newly identified markers can improve tumor cell detection without increasing off-target staining on PBMCs. Moreover, combining the new and known markers into a cocktail has the best potential to maximize coverage of diverse CTC subpopulations.

### New CTC markers improve CTC detection in TNBC mouse models

Having characterized the four new surface markers using cell lines, we next sought to determine their coverage on CTC samples from multiple mouse models. For these experiments, the tail vein injection method was used to simulate metastasis as it yields high numbers of CTCs after initial implantation in the lungs (39). We first validated the CTC markers using blood from the LM2-GFP model, which was originally used to produce our scRNA-seq CTC dataset. After PBMC enrichment, blood samples from these mice were stained with each of the four new markers individually (Supplementary Fig. S5A). GFP was used to distinguish CTCs from PBMCs. Similar to the *in vitro* results, the four individual markers only stained CTCs with variable intensity but did not show any off-target staining on PBMCs. Next, to assess differences in coverage between known and new markers, blood samples from LM2 mice were stained with cocktails containing either the published markers (denoted as published) or new and published markers combined (denoted as Total). Consistent with the cell line staining, LM2 CTCs showed little-to-no staining with the published cocktail; in contrast, the total cocktail showed a remarkable improvement in coverage (Supplementary Fig. S5B).

**Figure 5.**
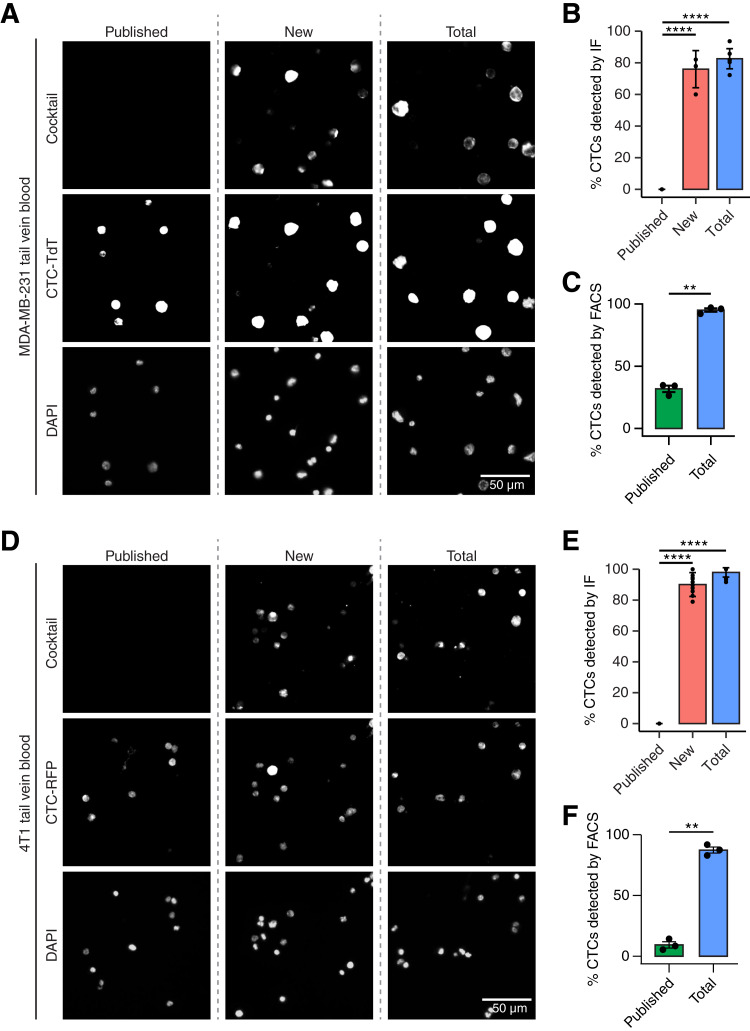
Combining known and novel surface markers expands CTC detection in mice. **A–C,** Data from CTCs isolated from the MDA-MB-231 tail vein injection mouse model. Representative images (**A**) and quantification (**B**) of TdTomato-tagged CTCs (middle column) immunofluorescently stained with published, new, or total cocktail for CTC surface markers (right column). **C,** Quantification of flow cytometry data for CTCs stained with published vs. total cocktail of CTC surface markers. **D–F,** Data from CTCs isolated from 4T1 tail vein injection mouse models. Representative images (**D**) and quantification (**E**) of RFP-tagged CTCs (middle column) immunofluorescently stained with published, new, or total cocktail for CTC surface markers (right column). **F,** Quantification of flow cytometry data for CTCs stained with published vs. total cocktail of CTC surface markers. For quantification in **B** and **E**, data are represented as mean ± SD, with *n* > 4 images analyzed per condition. For quantification in **C** and **F**, data are represented as mean ± SD, with *n* = 3 mice analyzed per model. See Supplementary Fig. S5D and S6E for flow cytometry gating strategy. DAPI served as a nuclear counterstain. ** indicates *P* < 0.01 and **** indicates *P* < 0.0001 using one-way ANOVA in **B** and **E** and two-tailed paired student *t* test in **C** and **F**.

We subsequently utilized two additional TNBC models, MDA-MB-231 and 4T1, to examine whether the new markers can successfully detect CTCs from a variety of backgrounds. MDA-MB-231 (231) is the parental cell line to LM2. When using IF staining, the coverage and signal for 231 CTCs were very poor with the published cocktail alone (Fig. 5A and B). Although some of these CTCs display dim signals with extremely high saturation settings, this dim staining is only barely above the background noise (Supplementary Fig. S5C) and thus would not allow confident identification of rare CTCs. In stark contrast, the new and total cocktails drastically improve the signal and coverage to 76% and 82.6%, respectively (Fig. 5A and B). As an alternative approach, we utilized flow cytometry to directly compare the effectiveness of the published versus total cocktails for detecting CTCs in blood from the same cohort of mice. Typical CTC counts are often too low to be reliably detected by flow cytometry. However, lung-metastatic tumors induced by tail vein injections generate higher numbers of CTCs, enabling characterization of staining patterns across thousands of CTCs in a single blood sample. For this analysis, CTCs were defined as the TdTomato^+^/CD45^−^ population (Supplementary Fig. S5D). As this method is more sensitive than IF staining, 40% of 231 CTCs were detected using the published cocktail (Fig. 5C), comparable with the CTCs detected via IF with high saturation settings (Supplementary Fig. S5C). Remarkably, the optimized total cocktail greatly expanded CTC detection to 95.2% (Fig. 5C).

Next, we examined the performance of these CTC markers in a metastatic mouse TNBC cell line, 4T1, as no existing markers can capture 4T1 CTCs. 4T1 is labeled with RFP and injected in mice via tail vein. Analysis of 4T1 CTCs isolated from the blood of tumor-bearing mice showed little-to-no detection with the published cocktail (Fig. 5D and E). In contrast, the new and total cocktails showed a remarkable increase in the detection of 4T1 CTCs to 90% and 97.9%, respectively (Fig. 5D and E). Consistent with the IF results, flow cytometry analysis showed a fourfold increase in the detection of the same CTCs with the total cocktail (Fig. 5F; Supplementary Fig. S5E). Overall, these findings demonstrate that traditional surface markers alone provide poor coverage and signal on these mouse models of metastatic TNBC. Combining the four new markers with the published markers significantly improves the detection of CTCs from diverse breast cancer cell lines and mouse models.

### A combinatorial staining approach improves detection of live CTCs in PDX and patient samples

**Figure 6.**
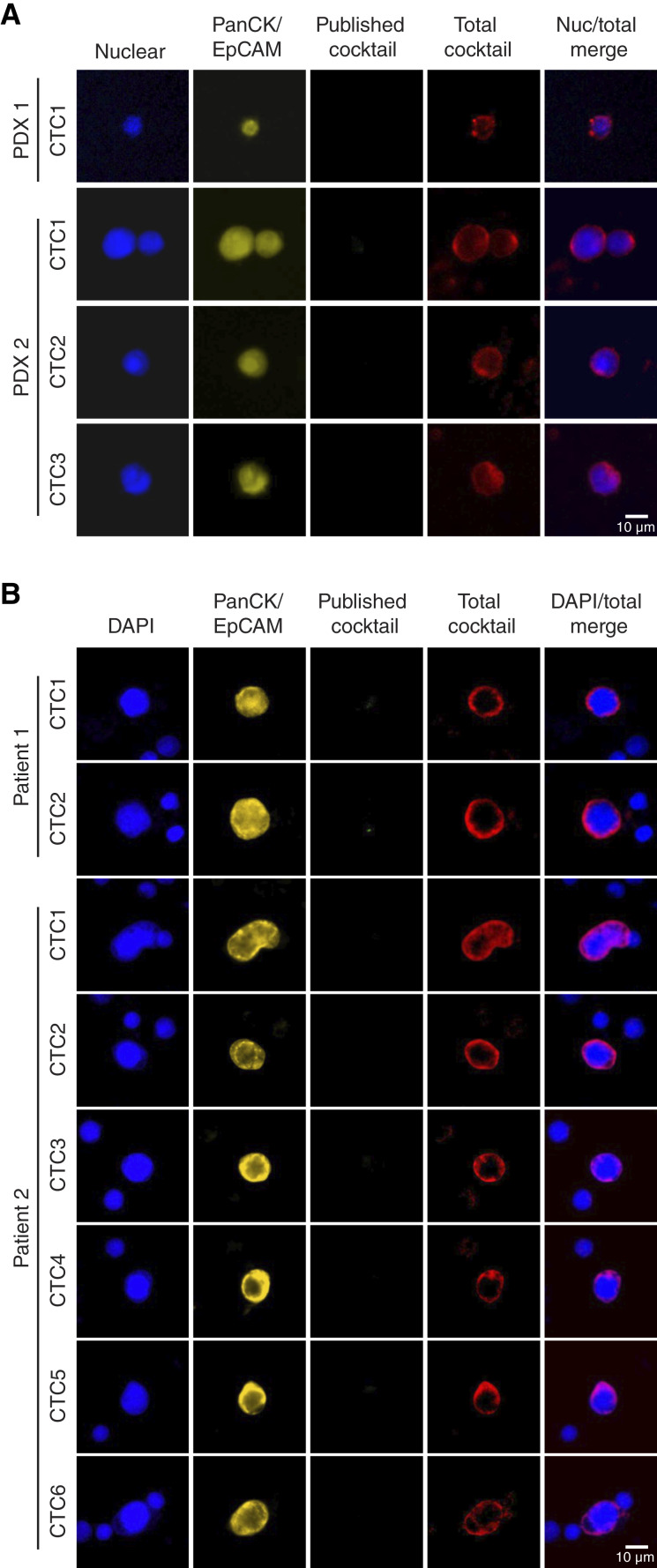
Novel surface marker cocktail expands CTC detection in PDX and patients. **A,** Representative images of blood samples from breast cancer PDX mice stained for hnRNPM (nuclear marker; blue), PanCK/EpCAM (yellow), published cocktail (green), and total cocktail (red). Merge of hnRNPM and total cocktail shown on right. **B,** Representative images of blood samples from patients with breast cancer stained for DAPI (nuclear marker; blue), PanCK/EpCAM (yellow), published cocktail (green), and total cocktail (red). Merge of DAPI and total cocktail shown on right.

Although well established, cell line–based models of metastasis have inherent limitations for faithful recapitulation of natural CTC biology. PDX models, which consist of patient tumors that have only been propagated in mice, more faithfully recapitulate the tumor characteristics and progression in patients than cell line–derived mouse models (40–43). Thus, PDX models likely produce CTCs with similar properties and heterogeneity to those found in patients. To further assess the effectiveness of the new CTC markers, we directly compared CTC detection by the published versus total cocktails via IF in blood samples from two TNBC PDX models, BCM-3904 and BCM-2665. As a note, the nuclear protein hnRNPM was used to stain nuclei instead of DAPI because of channel limitations (see Supplementary Fig. S6A for validation). As these PDX tumors lack a fluorescent tag, the standard combination of cytokeratin (CK) and EpCAM staining was used to identify CTCs. Blood isolated from PDX tumor-bearing mice was stained and detected in four channels: nuclear marker, PanCK/EpCAM, published cocktail, and total cocktail. The imaging results showed that the PDX-derived CTCs were negative for the published cocktail but distinctly detected with the total cocktail (Fig. 6A). Thus, CTCs from patients with TNBC can be missed when using current CTC surface markers (EpCAM, HER2, and EGFR) but can be readily detected by incorporating our new markers.

The above results were further recapitulated by the analysis of CTCs from two patients with TNBC. In both patient samples, none of the CK-positive CTCs displayed a positive signal for the published cocktail (Fig. 6B). However, these CTCs were clearly detected with the total cocktail (Fig. 6B). To ensure that the lack of positive staining in the channel for the published cocktail was not due to technical problems, we stained cell lines using the same protocol. As expected, the published cocktail robustly stained MCF7 cells and not the mesenchymal WHIM12 cells (Supplementary Fig. S6B), indicating that the published cocktail indeed fails to detect CTCs from samples of patients with TNBC. Interestingly, CK staining on WHIM12 cells is extremely dim (Supplementary Fig. S6B). These unknown features of patient CTCs emphasize the need for a combinatorial approach to identifying CTCs in the complex background of patient blood samples. We also found that the new surface markers are detected across cancer types at both the RNA and protein levels in CCLE data and across TCGA cancer projects (Supplementary Fig. S6C–S6E). Taken together, these results demonstrate that the total cocktail provides the best overall coverage, particularly for mesenchymal tumor cells. Of note, although cytokeratin staining can positively identify CTCs, which confirms the expression of our target markers, this method requires cell permeabilization and compromises nucleic acid integrity, precluding downstream transcriptomic analyses. Our described cocktail will help enhance the detection of diverse CTC subpopulations, facilitating better isolation and molecular characterization of live CTCs in the future.

## Discussion

The present study aimed to enhance the detection of live CTCs in breast cancer, particularly focusing on TNBC. We carefully selected methodologies meant to enhance our ability to molecularly characterize this rare cell population, which is important for an in-depth study of the metastatic process. This led us to develop a robust workflow for agnostic, marker-free identification and isolation of live CTCs for high-quality scRNA-seq. CTCs from an established TNBC model of metastasis demonstrate how heterogeneous this population of cells truly is. This dataset allowed us to reveal the gap in coverage for live CTC staining and find potential markers to improve identification of diverse CTC populations.

Our results highlight the limitations of relying on existing markers such as EpCAM for the detection of CTCs in blood samples. We identified new markers for CTCs, improving signal and coverage compared with traditional markers. The use of the combination of known and newly identified markers improved the detection rates of CTCs in TNBC while avoiding the use of cytokeratin. Importantly, the additional markers not only increase the rate of detection but also avoid off-target staining on PBMCs. The inclusion of marker-free depletion of immune cells by microfluidics also greatly increases the chance of finding rare cells in the retained fraction without creating a bias toward epithelial CTCs. PBMCs can be counter stained with CD45 for negative selection, further enhancing CTC detection accuracy. Our findings demonstrate advancements in the identification of live CTCs especially in TNBC in which cell characteristics vary broadly with no effective markers.

Detection via surface markers alone improves the ability to identify and isolate live cells and leads to better scRNA-seq quality for further study (6, 8, 9). The improved detection of live CTCs via surface markers is crucial for understanding the metastatic process and has the potential to identify targets for new therapies. Characterization of these surface proteins and the role they play in metastasis could lead to further discoveries, including metastatic mechanisms and markers of metastatic homing or tropism. Our findings emphasize the importance of using a comprehensive approach to identify and study CTCs and provide insights into the heterogeneity of tumor cells. PDX models closely mirror patient tumors (40–43) and likely produce CTCs with similar properties, enhancing the relevance of the findings to actual patient conditions. The new CTC markers each have connections to cancer and metastasis, including breast and lung cancers. For instance, AHNAK2 and CAVIN1 have been linked to proliferation and migration in different cancers (44–46) and AHNAK2 and ODR4 were identified as potential biomarkers for adenocarcinomas (47, 48). Also, AHNAK2 and TRIML2 were identified separately as potential prognostic biomarkers in TNBC (49, 50). A common site of breast cancer metastasis is the lungs, and our model systems generally have a high rate of lung metastasis at terminal collection. AHNAK2, CAVIN1, and ODR4 have all been linked to metastatic processes in lung adenocarcinomas (47, 48, 51) or non–small cell lung cancer (52), pointing to a potential overlap in functionality for homing or microenvironment adaptations.

We noted that some cytokeratin-positive patient CTCs did not stain with either cocktail, indicating the need to expand the markers used for surface-based identification of this population. Although cytokeratin staining may confirm the presence of CTCs, which we used to validate marker expression in our PDX and patient samples, it compromises nucleic acid integrity, limiting further molecular characterizations. The discovery of new markers in this study allows for live capture of intact CTCs, holding promise for downstream genomic and transcriptomic studies. Using the workflow described here, future studies can identify additional markers to capture the full heterogeneity of CTCs, which would provide a more comprehensive understanding of tumor biology and the metastatic cascade. In addition, this approach can be applied to other types of cancer, broadening the impact of CTC biology.

## Supplementary Material

Supplementary Fig. 1 Scatter plot and gene body coverage detailed plots.

Supplementary Fig. 2 Breakdown of previously published marker expression

Supplementary Fig. 3 Expression of potential CTC markers in breast cancer data

Supplementary Fig. 4 Potential CTC marker staining on PBMCs

Supplementary Fig. 5 Marker cocktails in flow data gating strategy

Supplementary Fig. 6 New CTC markers in breast cancer data

Supplementary Table 1 Reagents used for CTC processing and SMARTseq

Supplementary Table 2 List of antibodies used with catalog and RRID numbers

Supplementary Table 3 List of RRIDs for cell lines, plasmids and software packages

## Data Availability

The data generated in this study are publicly available in GEO as GSE295462. All code used in this study and all other data are available upon request from the corresponding author.
