# Supplementary material for: Surface Marker Identification to Capture Live Circulating Tumor Cells in Metastatic Triple-Negative Breast Cancer
Source: Cancer Res Commun. 2026 Jan 15;6(1):115–29. doi: 10.1158/2767-9764.CRC-25-0536 (PMC12805936; doi:10.1158/2767-9764.CRC-25-0536)
Supplement: Supplementary Fig. 1 — Scatter plot and gene body coverage detailed plots. [file crc-25-0536_supplementary_fig.1_suppsf1.pdf]

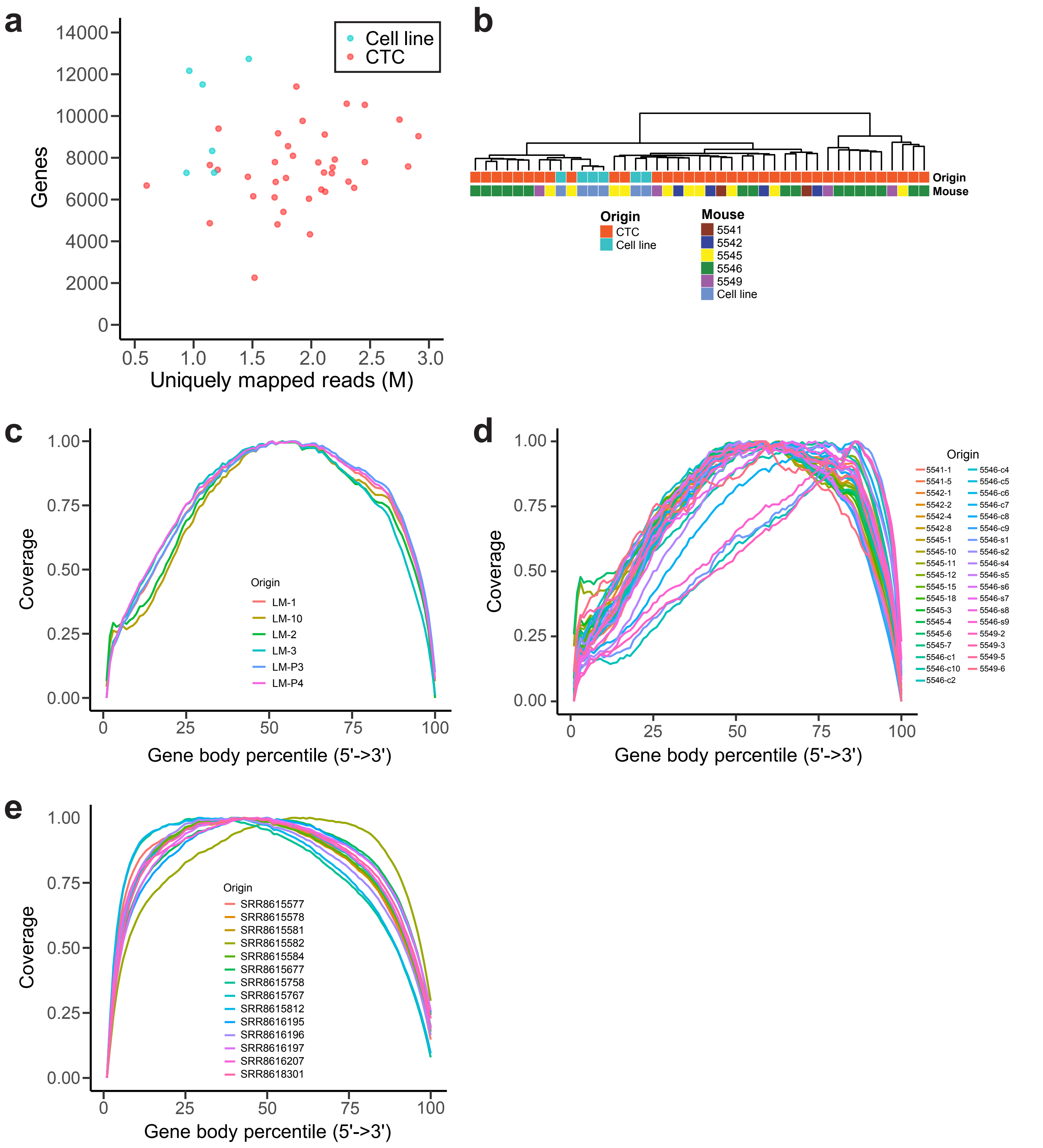
a
Genes
Uniquely mapped reads (M)
Cell line
CTC
0
2000
4000
6000
8000
10000
12000
14000
0.5
1.0
1.5
2.0
2.5
3.0
b
Origin
Mouse
Origin
CTC
Cell line
Mouse
5541
5542
5545
5546
5549
Cell line
c
Coverage
Gene body percentile (5'->3')
Origin
LM-1
LM-10
LM-2
LM-3
LM-P3
LM-P4
0.00
0.25
0.50
0.75
1.00
0
25
50
75
100
d
Coverage
Gene body percentile (5'->3')
Origin
5541-1
5541-5
5542-1
5542-2
5542-4
5542-8
5545-1
5545-10
5545-11
5545-12
5545-15
5545-18
5545-3
5545-4
5545-6
5545-7
5546-c1
5546-c10
5546-c2
5546-c4
5546-c5
5546-c6
5546-c7
5546-c8
5546-c9
5546-s1
5546-s2
5546-s4
5546-s5
5546-s6
5546-s7
5546-s8
5546-s9
5549-2
5549-3
5549-5
5549-6
e
Coverage
Gene body percentile (5'->3')
Origin
SRR8615577
SRR8615578
SRR8615581
SRR8615582
SRR8615584
SRR8615677
SRR8615758
SRR8615767
SRR8615812
SRR8616195
SRR8616196
SRR8616197
SRR8616207
SRR8618301

Supplementary Fig. 1 SMARTseq2 scRNAseq from CTCs has similar gene-body coverage to bulk RNAseq. (a) Scatter plot of Gene count versus Uniquely mapped reads (M) (**b)** Dendrogram representation for hierarchical clustering of sample correlation heatmap displayed in Figure 1d. **c-e.** Meta-coverage plots showing gene body coverage for individual LM2 CTCs (**c**), LM2 cell line cells (**d**), and CCLE breast cancer cell lines (**e**). See Figure 1f for averaged plots.
