# Supplementary material for: Surface Marker Identification to Capture Live Circulating Tumor Cells in Metastatic Triple-Negative Breast Cancer
Source: Cancer Res Commun. 2026 Jan 15;6(1):115–29. doi: 10.1158/2767-9764.CRC-25-0536 (PMC12805936; doi:10.1158/2767-9764.CRC-25-0536)
Supplement: Supplementary Fig. 2 — Breakdown of previously published marker expression [file crc-25-0536_supplementary_fig.2_suppsf2.pdf]

Supplementary Fig. 2

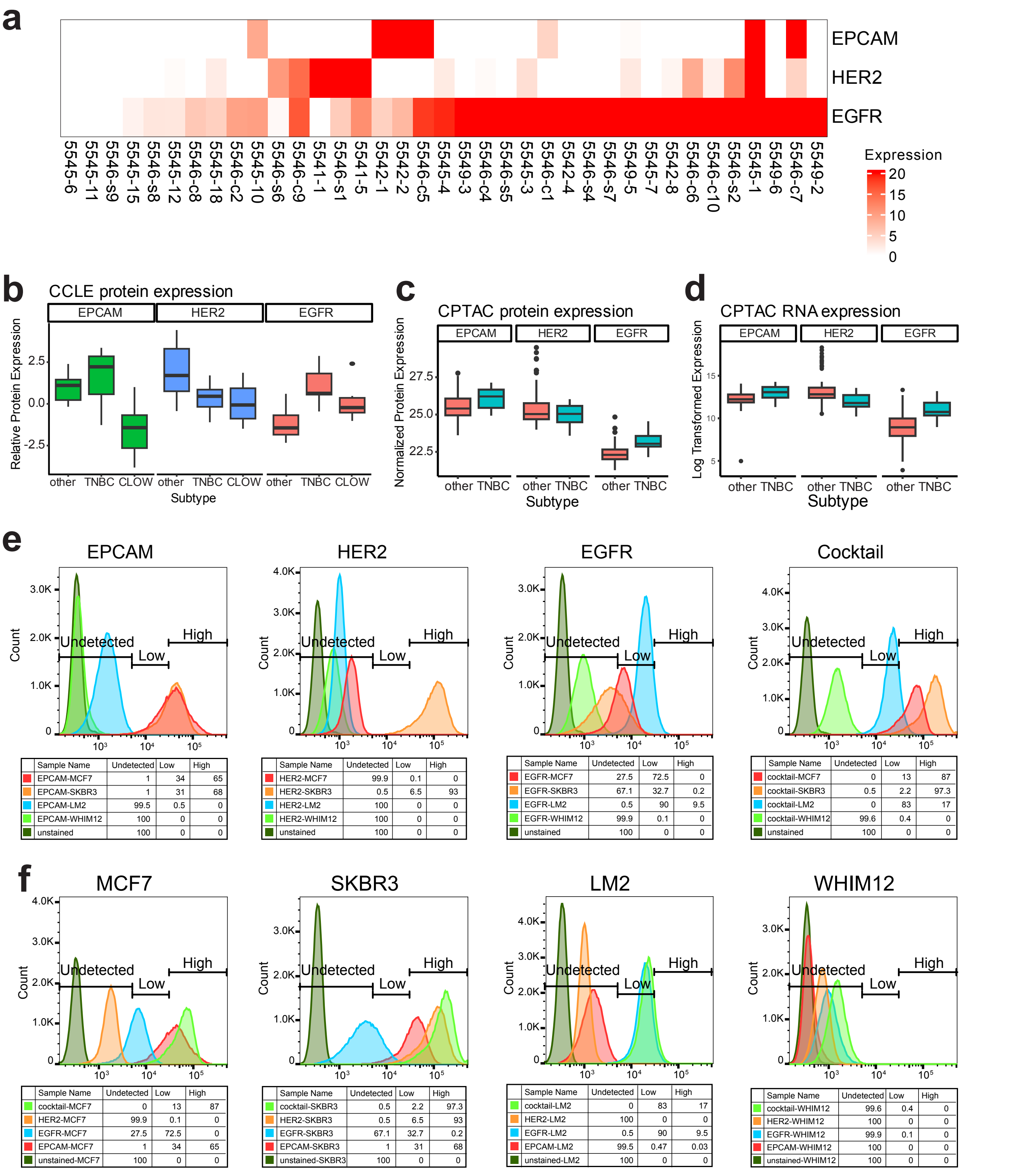

	Sample Name	Undetected	Low	High
	EPCAM-MCF7	1	34	65
	EPCAM-SKBR3	1	31	68
	EPCAM-LM2	99.5	0.5	0
	EPCAM-WHIM12	100	0	0
	unstained	100	0	0

	Sample Name	Undetected	Low	High
	HER2-MCF7	99.9	0.1	0
	HER2-SKBR3	0.5	6.5	93
	HER2-LM2	100	0	0
	HER2-WHIM12	100	0	0
	unstained	100	0	0

	Sample Name	Undetected	Low	High
	EGFR-MCF7	27.5	72.5	0
	EGFR-SKBR3	67.1	32.7	0.2
	EGFR-LM2	0.5	90	9.5
	EGFR-WHIM12	99.9	0.1	0
	unstained	100	0	0

	Sample Name	Undetected	Low	High
	cocktail-MCF7	0	13	87
	cocktail-SKBR3	0.5	2.2	97.3
	cocktail-LM2	0	83	17
	cocktail-WHIM12	99.6	0.4	0
	unstained	100	0	0

	Sample Name	Undetected	Low	High
	cocktail-MCF7	0	13	87
	HER2-MCF7	99.9	0.1	0
	EGFR-MCF7	27.5	72.5	0
	EPCAM-MCF7	1	34	65
	unstained-MCF7	100	0	0

	Sample Name	Undetected	Low	High
	cocktail-SKBR3	0.5	2.2	97.3
	HER2-SKBR3	0.5	6.5	93
	EGFR-SKBR3	67.1	32.7	0.2
	EPCAM-SKBR3	1	31	68
	unstained-SKBR3	100	0	0

	Sample Name	Undetected	Low	High
	cocktail-LM2	0	83	17
	HER2-LM2	100	0	0
	EGFR-LM2	0.5	90	9.5
	EPCAM-LM2	99.5	0.47	0.03
	unstained-LM2	100	0	0

	Sample Name	Undetected	Low	High
	cocktail-WHIM12	99.6	0.4	0
	HER2-WHIM12	100	0	0
	EGFR-WHIM12	99.9	0.1	0
	EPCAM-WHIM12	100	0	0
	unstained-WHIM12	100	0	0

Supplementary Fig. 2 Previously published CTC surface markers lack coverage in TNBC subtypes. **(a)** Heatmap of normalized RNA expression for EpCAM, HER2, and EGFR from scRNAseq of LM2 CTCs (n=37). (**b)** Relative protein expression for EpCAM, HER2, and EGFR in CCLE Breast Cancer cell lines divided into claudin-low (CLOW), TNBC minus CLOW, and all other subtypes. (**c, d)** Normalized protein expression (**c**) and Log2 transformed RSEM RNA expression (**d**) for EpCAM, HER2, and EGFR in CPTAC Breast Cancer patient tumors, divided into TNBC and non-TNBC subtypes. Boxes indicate median and interquartile range; whiskers show minima and maxima, and dots indicate outliers. (**e, f)** Histogram gates for quantification of flow cytometry staining mean fluorescence intensity for EpCAM, HER2, and EGFR individually and together (Cocktail). Data for MCF7, SKBR3, LM2, and WHIM12 cell lines is shown, either grouped by marker(**e**) or cell line (**f**).
