# Supplementary material for: Surface Marker Identification to Capture Live Circulating Tumor Cells in Metastatic Triple-Negative Breast Cancer
Source: Cancer Res Commun. 2026 Jan 15;6(1):115–29. doi: 10.1158/2767-9764.CRC-25-0536 (PMC12805936; doi:10.1158/2767-9764.CRC-25-0536)
Supplement: Supplementary Fig. 3 — Expression of potential CTC markers in breast cancer data [file crc-25-0536_supplementary_fig.3_suppsf3.pdf]

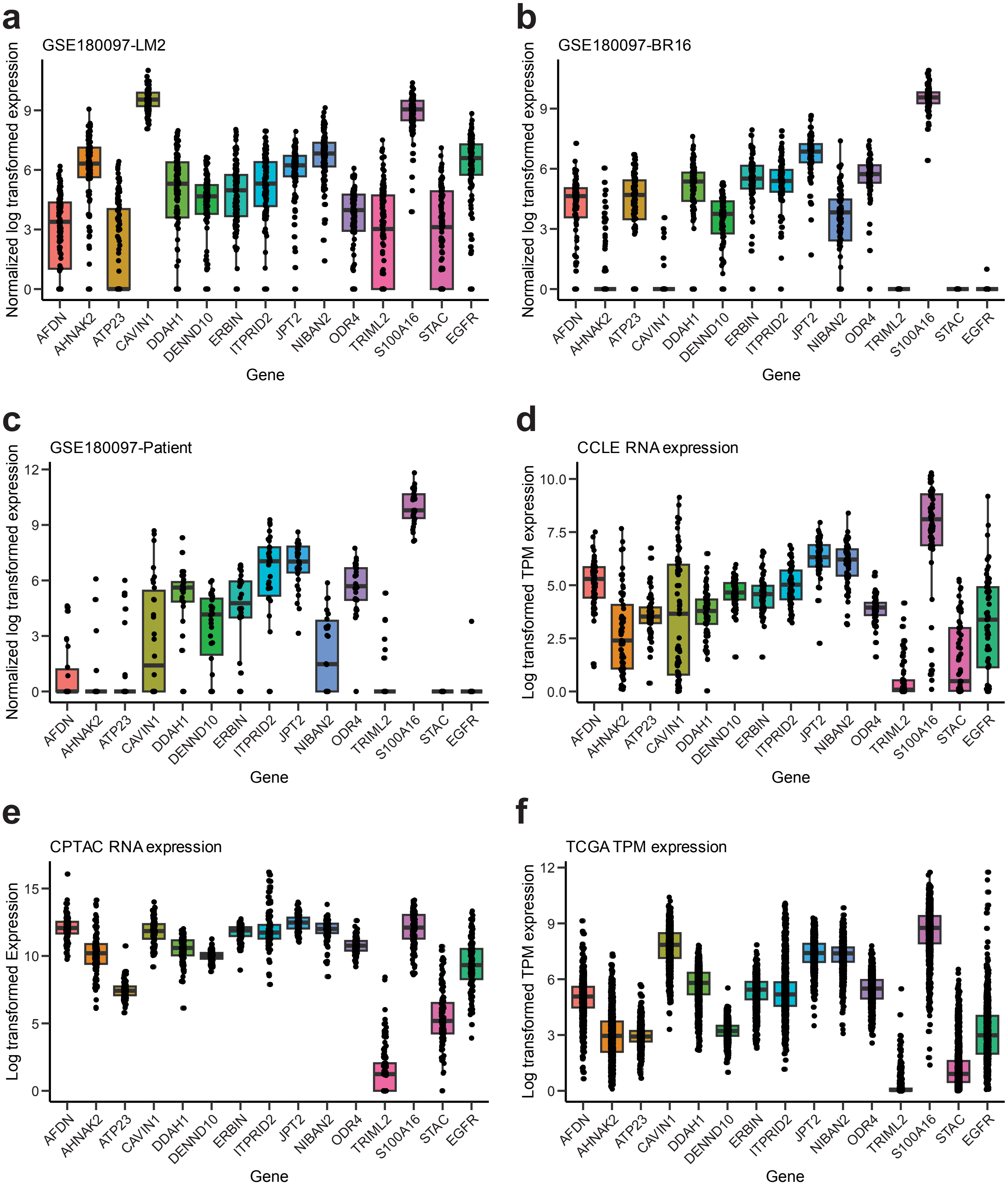
a
GSE180097-LM2
b
GSE180097-BR16
c
GSE180097-Patient
d
CCLE RNA expression
e
CPTAC RNA expression
f
TCGA TPM expression
Normalized log transformed expression
Log transformed TPM expression
Log transformed Expression
Gene
AFDN
AHNAK2
ATP23
CAVIN1
DDAH1
DENND10
ERBIN
ITPRID2
JPT2
NIBAN2
ODR4
TRIML2
S100A16
STAC
EGFR

Supplementary Fig. 3 Potential CTC surface markers appear in Breast Cancer data from multiple sources. **(a-c)** Normalized log transformed RNA expression of 14 potential CTC surface markers and known marker EGFR in CTCs from GSE109761, divided into LM2 (n=116) (**a**), BR16 (n=95) (**b**), and patient samples (n=30) (**c**). (**d-f)** Log transformed TPM RNA expression of 14 potential CTC surface markers and known marker EGFR in CCLE Breast Cancer cell lines (**d**), CPTAC Breast Cancer patient tumors (**e**), and TCGA Breast Cancer patient tumors (**f**). Boxes indicate median and interquartile range; whiskers show minima and maxima, and dots indicate outliers.
