# Supplementary material for: Surface Marker Identification to Capture Live Circulating Tumor Cells in Metastatic Triple-Negative Breast Cancer
Source: Cancer Res Commun. 2026 Jan 15;6(1):115–29. doi: 10.1158/2767-9764.CRC-25-0536 (PMC12805936; doi:10.1158/2767-9764.CRC-25-0536)
Supplement: Supplementary Fig. 4 — Potential CTC marker staining on PBMCs [file crc-25-0536_supplementary_fig.4_suppsf4.pdf]

Supplementary Fig. 4

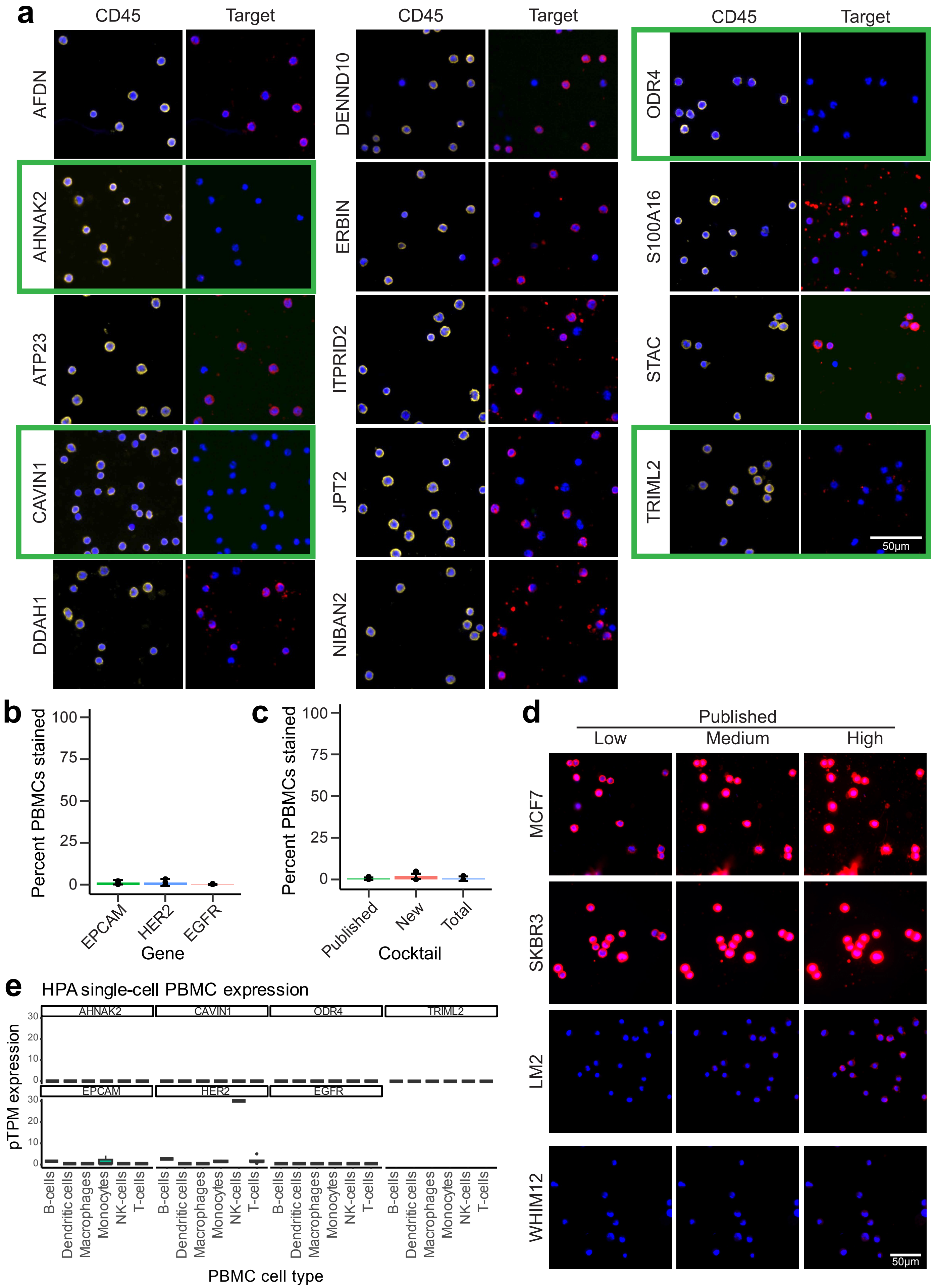

Supplementary Fig. 4 New marker selection avoids off-target staining on PBMCs. **(a)** Representative images of human PBMCs stained with CD45 (left columns; yellow) and CTC surface marker candidates (right columns; red). See Fig. 4b for quantification. (**b)** Quantification of percent positive staining for individual published markers on human PBMCs. (**c)** Quantification of percent positive staining on PBMCs for surface marker cocktails: Published (EpCAM, HER2, EGFR), New (AHNAK2, CAVIN1, ODR4, TRIML2), and Total (EpCAM, HER2, EGFR, AHNAK2, CAVIN1, ODR4, TRIML2). For quantification in **b, c**: data are represented as mean ± SD, n > 4 images analyzed per condition. (**d)** Representative images of cell lines stained with Published cocktail (EpCAM, HER2, EGFR; red). Images taken at low, medium and high saturation settings are shown. DAPI (blue) served as a nuclear counterstain. **(e)** Normalized (TPM) RNA expression of 3 published and 4 new CTC surface markers from HPA single cell data for PBMCs. Boxes indicate median and interquartile range; whiskers show minima and maxima, and dots indicate outliers.
