# Supplementary material for: Surface Marker Identification to Capture Live Circulating Tumor Cells in Metastatic Triple-Negative Breast Cancer
Source: Cancer Res Commun. 2026 Jan 15;6(1):115–29. doi: 10.1158/2767-9764.CRC-25-0536 (PMC12805936; doi:10.1158/2767-9764.CRC-25-0536)
Supplement: Supplementary Fig. 5 — Marker cocktails in flow data gating strategy [file crc-25-0536_supplementary_fig.5_suppsf5.pdf]

Supplementary Fig. 5

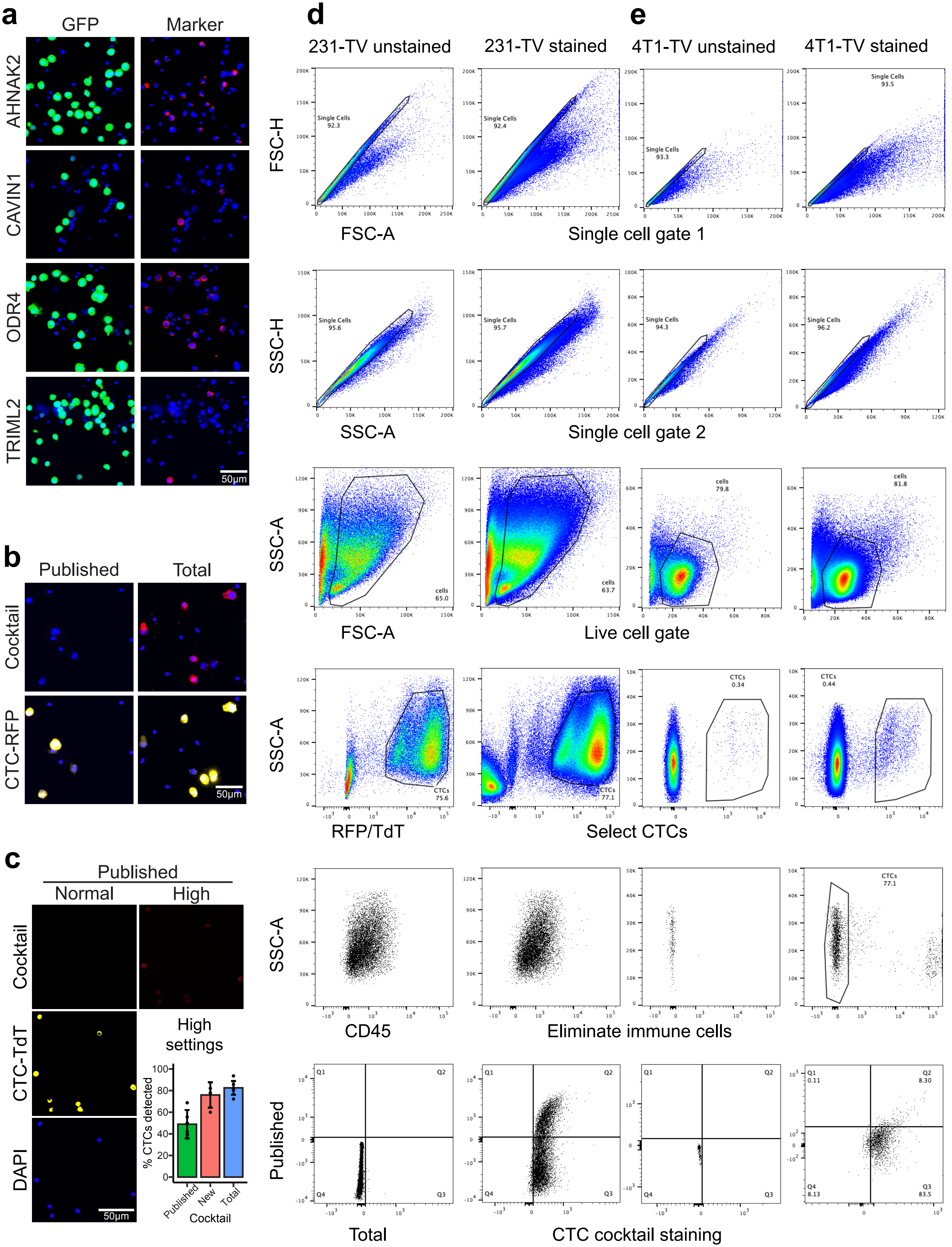

**Supplementary Fig. 5 Combined marker cocktail detects CTCs in multiple mouse models of TNBC**. **(a)** Immunofluorescent staining of LM2 tail vein blood samples with four new markers that have little or no staining on PBMCs and one marker with strong PBMC staining. GFP-tagged CTCs (left; green) and marker staining (right; red) shown. (**b)** Immunofluorescent staining of LM2 tail vein RFP-tagged CTCs (bottom row; yellow) with Published or Total cocktails (top row; red). (**c)** Immunofluorescent staining of 231 tail vein TdTomato-tagged CTCs (middle left; yellow) with published markers at normal settings (top left; red) and much higher saturation settings (top right; red). Bottom right panel shows quantification of published marker staining on 231 CTCs using higher saturation pictures. (**d, e)** Gating strategy for flow cytometry data for CTCs in 231 (**d**) and 4T1 (**e**) tail vein mice stained for Published versus Total cocktails. For quantification in **a**: data are represented as mean ± SD, n > 4 images analyzed per condition. DAPI served as a nuclear counterstain.
