# Supplementary material for: Surface Marker Identification to Capture Live Circulating Tumor Cells in Metastatic Triple-Negative Breast Cancer
Source: Cancer Res Commun. 2026 Jan 15;6(1):115–29. doi: 10.1158/2767-9764.CRC-25-0536 (PMC12805936; doi:10.1158/2767-9764.CRC-25-0536)
Supplement: Supplementary Fig. 6 — New CTC markers in breast cancer data [file crc-25-0536_supplementary_fig.6_suppsf6.pdf]

Supplementary Fig. 6

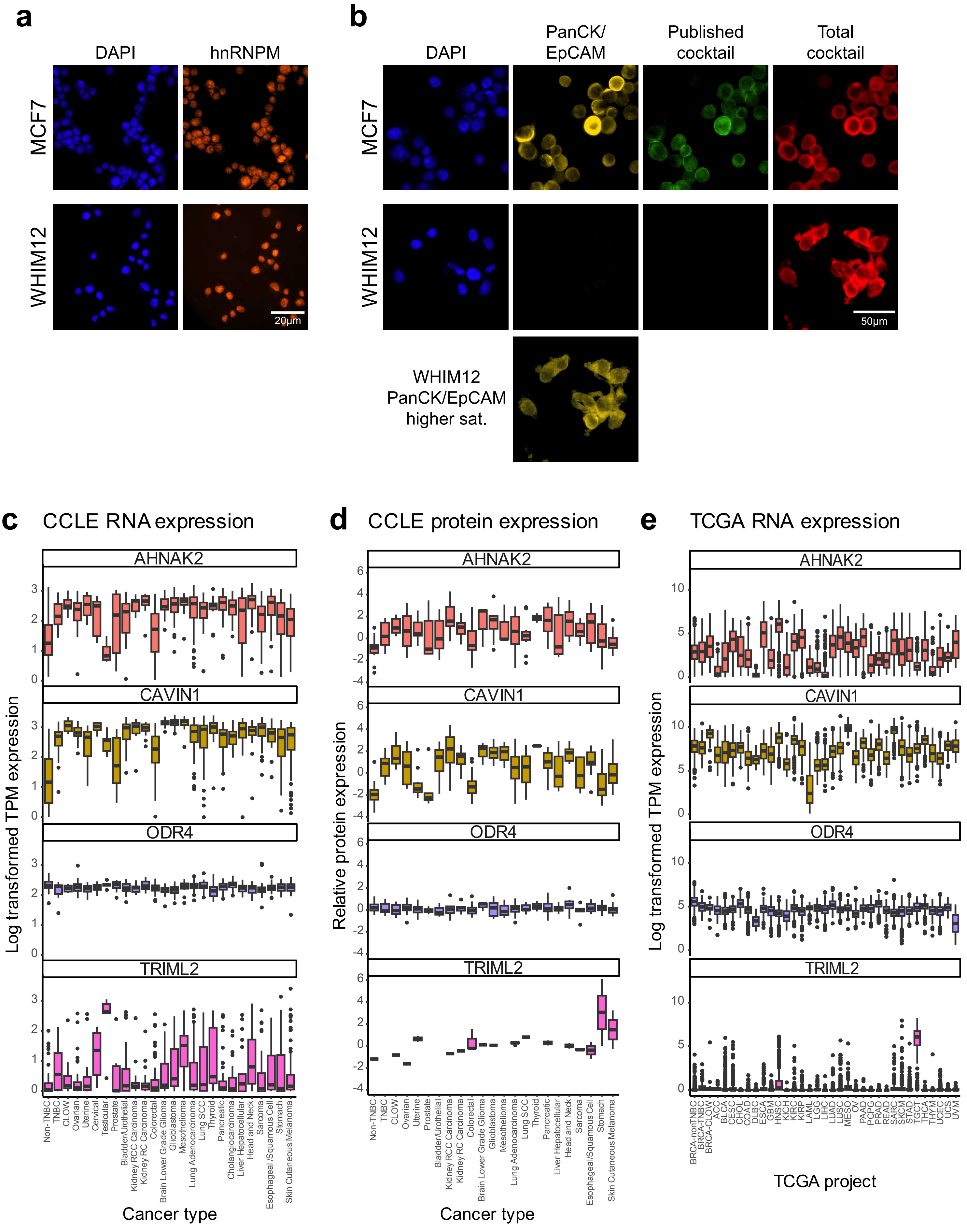

Supplementary Fig. 6 Novel surface marker cocktail expands CTC detection in PDX and patients. (**a)** Representative images of MCF7 and WHIM12 cell lines stained with DAPI (blue) and hnRNPM (orange), showing complete overlap of staining on nuclei. (**b)** Representative images of MCF7 and WHIM12 cell lines stained with DAPI (nuclear marker; blue), PanCK/EpCAM (yellow), Published cocktail (green), and Total cocktail (red). PanCK/EpCAM staining (yellow) for WHIM12 cells is shown at higher saturation settings below to display their dim positivity. **(c)** Log transformed TPM RNA expression of 4 new CTC surface markers in CCLE Cancer cell lines. **(d)** Relative protein expression of 4 new CTC surface markers in CCLE Cancer cell lines. **(e)** Log transformed TPM RNA expression of 4 new CTC surface markers in TCGA Cancer patient tumors. Boxes indicate median and interquartile range; whiskers show minima and maxima, and dots indicate outliers.
