# Supplementary material for: Surface Marker Identification to Capture Live Circulating Tumor Cells in Metastatic Triple-Negative Breast Cancer
Source: Cancer Res Commun. 2026 Jan 15;6(1):115–29. doi: 10.1158/2767-9764.CRC-25-0536 (PMC12805936; doi:10.1158/2767-9764.CRC-25-0536)
Supplement: Supplementary Table 1 — Reagents used for CTC processing and SMARTseq [file crc-25-0536_supplementary_table_1_suppst1.pdf]

Supplementary table 1 – Reagent list

**CTC processing**		
**EDTA tubes**	BD	365974
**EDTA 0.5M**	Thermo Fisher	AM9260G
**SMARTseq**		
**Lysis**		
**RNaseOUT**	Invitrogen	10-777-019
**Triton**	Sigma	93443-100mL
**dNTP**	Fisher Scientific	FERR0192
**Oligo**	IDT	AAGCAGTGGTATCAACGCAGAGTACTTTTTTTTTTTTTTTTTTTTTTTTTTTTTVN
**RT**		
**5xFS buffer**	Takara	in Smartscribe
**RNase OUT**	Invitrogen	10-777-019
**DTT**	Takara	in smartscribe
**MgCl2**	Thermo Fisher	AM9530G
**Betaine**	Sigma-Aldrich	B0300-5VL
**TSO**	Exiqon	TSO (100uM) LNA primer SMARTSeq2_TSO_LNA 5'-AAGCAGTGGTATCAACGCAGAGTACATrGrG+G
**Smartscribe RT**	Takara	639538
**Amplification**		
**IS PCR primer**	IDT	AAGCAGTGGTATCAACGCAGAGT
**KAPA HiFi hotstart**	Roche	7958935001
**Lambda exonuclease**	NEB	M0262L
