# Supplementary material for: Surface Marker Identification to Capture Live Circulating Tumor Cells in Metastatic Triple-Negative Breast Cancer
Source: Cancer Res Commun. 2026 Jan 15;6(1):115–29. doi: 10.1158/2767-9764.CRC-25-0536 (PMC12805936; doi:10.1158/2767-9764.CRC-25-0536)
Supplement: Supplementary Table 2 — List of antibodies used with catalog and RRID numbers [file crc-25-0536_supplementary_table_2_suppst2.pdf]

Supplementary table 2 – Antibody list

Marker	Supplier	Clone	Blue	AF488	PE/AF568	AF647	Unconjugated
**Hoechst**	Thermo Fisher		62249				
**DAPI**	Boster		AR1176				
**EpCAM**	Cell Signaling	VU1D9		5198S, RRID: AB_10692105	7319, RRID: AB_10949504	5447S, RRID: AB_10693458	2929S, RRID: AB_2098657
**HER2**	Biolegend	24D2		324410, RRID: AB_2099256	324406, RRID: AB_756122	324412, RRID: AB_2262300	324402, RRID: AB_756118
**EGFR**	Biolegend	AY13		352907, RRID: AB_11124324	352916, RRID: AB_2563210	352905, RRID: AB_11148943	352902, RRID: AB_10945161
**PanCK**	Santa Cruz	C11					sc-8018, RRID: AB_627396
**CK19**	Invitrogen						MA5-12663, RRID: AB_10984317
**CD45-h**	Invitrogen	2D1			12-9459-41, RRID: 10732347		
**CD45-m**	BD	30-F11			553081, RRID: AB_394611		
**AHNAK2**	Sino Biological						203703-T10, RRID: AB_3713543
**CAVIN1 (PTRF)**	Sigma						HPA074213, RRID: AB_2686674
**ODR4 (C1orf27)**	Sino Biological						204562-T02, RRID: AB_3713544
**TRIML2**	Sigma						HPA043838, RRID: AB_10963835
**AFDN**	Novus Biological						af7829, RRID: AB_3644507
**ATP23**	Sigma						HPA043204, RRID: AB_2678361
**DDAH1**	Sino Biological						102610-T10, RRID: AB_3713545
**DENND10 (FAM45A)**	Novus Biological						NBP1-86232, RRID: AB_11026016
**ERBIN**	Novus Biological						af7866, RRID: AB_3644512
**ITPRID2 (SSFA2)**	Sigma						HPA034665, RRID: AB_2674271
**JPT2 (HN1L)**	Sigma						HPA041908, RRID: AB_10794160
**NIBAN2 (FAM129B)**	Sino Biological						202505-T46, RRID: AB_3713546
**S100A16**	Sino Biological						11137-RP02, RRID: AB_3713547
**STAC**	Sino Biological						205604-T08, RRID: AB_3713548
**hnRNPM**	Bethyl	hnRNP M3/4					A303-910A, RRID: AB_2620260
**anti-Rabbit secondary**	Invitrogen			A21206, RRID: AB_2535792		A10931, RRID: AB_10562534	
**anti-Mouse secondary**	Invitrogen		A21049, RRID: AB_2535717		A11004, RRID: AB_2534072		
**anti-Sheep secondary**	Novus					NBP1-75670, RRID: AB_11009056	
