# Supplementary material for: Surface Marker Identification to Capture Live Circulating Tumor Cells in Metastatic Triple-Negative Breast Cancer
Source: Cancer Res Commun. 2026 Jan 15;6(1):115–29. doi: 10.1158/2767-9764.CRC-25-0536 (PMC12805936; doi:10.1158/2767-9764.CRC-25-0536)
Supplement: Supplementary Table 3 — List of RRIDs for cell lines, plasmids and software packages [file crc-25-0536_supplementary_table_3_suppst3.pdf]

Supplementary table 3 – Cell line, plasmid, and software list

**Experimental Models: Cell Lines**	
MDA-MB-231	RRID:CVCL_0062
MDA-MB-231-LM2	RRID: CVCL_A5ER
SKBR3	RRID:CVCL_0033
4T1	RRID: CVCL_0125
MCF7	RRID:CVCL_0031
**Recombinant DNA**	
pFUGW-tdtomato	RRID: Addgene_22478
pWPT-RFP	Dr. Xiang Zhang
**Software and Algorithms**	
Gencode	RRID: SCR_014966
STAR	RRID: SCR_004463
Pheatmap	RRID: SCR_016418
DESeq2	RRID:SCR_015687
GEO DataSets	RRID: SCR_005012
BioProject	RRID: SCR_004801
RSeQC	RRID: SCR_005275
ggplot2	RRID: SCR_014601
HPA	RRID: SCR_006710
Ensembl	RRID: SCR_006773
BioMart	RRID: SCR_019214
ImageJ	RRID:SCR_003070
FlowJo	RRID:SCR_008520
